# Exosomal circSCMH1/miR-874 ratio in serum to predict carotid and coronary plaque stability

**DOI:** 10.3389/fcvm.2023.1277427

**Published:** 2023-12-11

**Authors:** Jiayu Wang, Yixuan Liu, Peiqing Tian, Liyun Xing, Xianwei Huang, Caihua Fu, Xiangyu Xu, Ping Liu

**Affiliations:** ^1^ Department of Cardiology, The Second Hospital of Shandong University, Shandong, China; ^2^ Tianjin Key Laboratory of Ionic-Molecular Function of Cardiovascular Disease, Department of Cardiology, Tianjin Institute of Cardiology, The Second Hospital of Tianjin Medical University, Tianjin, China; ^3^ Division of Cardiology, Beijing Anzhen Hospital, Capital Medical University, Beijing, China; ^4^ Department of Emergency, The First Affiliated Hospital of Xiamen University, Fujian, China; ^5^ Department of Cardiology, Jinan Central Hospital Affiliated Shandong University, Shandong, China

**Keywords:** exosomes, non-coding RNAs, acute coronary syndrome, plaque stability, coronary artery disease

## Abstract

**Background:**

To investigate the correlation between lg (circSCMH1/miR-874) and acute coronary syndrome (ACS), acute myocardial infarction (AMI), and carotid plaque stability.

**Methods:**

701 patients were divided into stable coronary artery disease (SCAD), ACS, and control groups. Furthermore, 225 patients who underwent carotid ultrasound were selected from the above 701 patients and were divided into low-risk plaque, medium-to-high risk plaque, and control (without carotid plaques) groups. We collected their baseline characteristics and measured the contents of exosomal circSCMH1 and miR-874 in peripheral blood. Then lg(circSCMH1/miR-874) was calculated and statistical analysis was performed.

**Results:**

The lg (circSCMH1/miR-874) values of ACS, SCAD, and the control group decreased successively (*P* < 0.05). Compared with the low-risk plaque and control groups, the lg (circSCMH1/miR-874) value of medium-high risk plaque group decreased (*P* < 0.05). Multivariate logistic regression analysis showed that with the decrease of lg (circSCMH1/miR-874), the risk of ACS, AMI, and medium-high risk plaques increased. ROC curve analysis demonstrated that lg (circSCMH1/miR-874) has a higher diagnostic value for ACS, AMI and medium-high risk plaques than previously used predictive ratios.

**Conclusion:**

Lg (circSCMH1/miR-874) is closely associated with coronary and carotid plaque stability.

## 1. Introduction

Arteriosclerotic cardiovascular disease (ASCVD), especially ischemic heart disease and ischemic stroke, is the first killer threatening the health of World population (1). The prevalence of cardiovascular disease has almost doubled from 271 million cases in 1990 to 523 million cases in 2019. Deaths from cardiovascular disease have steadily increased from 12.1 million in 1990 to 18.6 million in 2019. China has the highest number of deaths resulting from cardiovascular disease (1). Multiple factors can cause arterial endothelial injury and lipid deposition, leading to atherosclerosis (AS), which can involve the large and middle arteries of the whole body (2). When the atherosclerotic plaques rupture or become unstable, it becomes an important cause of ischemic heart disease and stroke (2). Patients with carotid or peripheral arterial disease (PAD) usually have extensive atherosclerosis, higher incidences of multiple coronary and cerebral arterial thrombotic events, and higher mortalities (2, 3).

Coronary artery plaque is the main pathological manifestation of coronary atherosclerosis, and the stability of plaque directly affects the clinical symptoms, management principles, therapeutic effects, and prognosis of coronary heart disease (4). Unstable plaque in the intima of the coronary artery can lead to thrombosis and cause life-threatening diseases such as acute myocardial infarction (AMI), heart failure, malignant arrhythmia, and sudden death (4). Patients with acute coronary syndrome (ACS) have significantly different atherosclerosis phenotypes, including thinner fibrous cap of plaque and lower plaque healing rate, compared to the patients with stable coronary heart disease (4, 5).

Exosomes are vesicles with a diameter of 30–150 nm containing a lipid bilayer structure (6, 7). Due to this structure, exosomes naturally serve as carriers that can transfer non-coding ribonucleic acids (ncRNAs) between cells and tissues, including microRNA (miRNA), long non-coding RNA (LncRNA), and circular RNA (circRNA) (6, 7). Exosomes can fulfill a vital role in the transmission of biological information and have a wide range of applications in cardiovascular diseases. MiRNAs are the most studied component of exosomes because they play a significant role as novel biomarkers in exosomes, and some miRNAs have been used to predict cardiovascular diseases such as AMI (7). CircRNA has a circular structure that better resists nuclease degradation, making it more stable than linear RNA molecules, and becomes a potential biomarker in the blood of patients with certain diseases (6–9). However, whether peripheral blood circRNAs can reflect or predict the stability of carotid and coronary plaque has not been reported.

Previous studies have shown that factors such as hypertension, diabetes, and dyslipidemia are related to the formation of atherosclerotic plaques, and various indicators or ratios have been proposed, such as neutrophil/lymphocyte ratio (NLR) (10), monocyte/ lymphocyte (MLR), uric acid/neutrophil, monocyte/HDL-C, triglyceride/HDL-C ratios (11–13), peripheral blood platelet/lymphocyte ratio (PLR) (14), homocysteine (HCY), lipoprotein-associated phospholipase A2, retinol-binding protein, and miRNAs such as miR-21, miR-34a, miR-126, miR-155, and miR-221. However, these indicators have some drawbacks, such as poor sensitivity, low specificity, weak stability, and susceptibility to environmental influences (10, 12, 15). To estimate and predict the incidence of coronary events, imaging examinations such as coronary computed tomography angiography (CCTA), coronary angiography (CAG), intravascular ultrasound imaging (IVUS), optical coherence tomography (OCT), etc., are not only time-consuming and expensive but also cause adverse reactions such as contrast agent allergy and contrast agent nephropathy (16).

Non coding RNA (ncRNA) has become an important effector of intimal thickening and a potential circulating biomarker of cardiovascular disease (17). CircSCMH1 is a circular RNA that has been found to be significantly reduced in models of ischemic stroke (18). Recent studies have shown that circSCMH1 can increase the ubiquitination-modified fat mass and obesity-associated protein (FTO) translocation to endothelial cell nucleus, leading to the N6-methyladenosine (m6A) demethylation of phospholipid phosphatase 3 (Plpp3) mRNA, thereby enhancing its expression and promoting vascular repair after stroke (19). Therefore, we sought to explore whether circSCMH1 plays a similar role in vulnerable plaque and hoped to explore its network of action.

To search for target RNAs and speculate on possible interaction networks, we applied the method of bioinformatics analysis to search the literature and query the public RNA interaction database (Fig. http://www.mirdb.org, circinteractome.irp.nia.nih.gov, et al.) (Supplementary Table S1). After preliminary experiments, we chose hsa-miR-874 as the target miRNA.

Hsa-miR-874 (abbreviated as miR-874) has been found to have altered expression levels in various tumors and to have a protective effect on the kidneys in diabetic nephropathy (20–25). In terms of myocardial injury, although some studies have shown that miR-874 is down-regulated in AMI patients (26), other studies have shown that miR-874 can promote apoptosis, inhibit cell proliferation, and accelerate vascular calcification (27, 28).

Previous studies have used peripheral blood circRNAs or miRNAs to predict the occurrence and severity of cardiovascular disease, but most of them are single-factor predictions, and no reports have been found on using the ratio of circRNAs to miRNAs to forecast the stability of atherosclerotic plaques. This study aims to simultaneously study circRNAs and miRNAs to explore the relationship between the ratio of circSCMH1 to miR-874 and the occurrence of ACS, AMI and high-risk plaques. Then we can explore whether this ratio can become a more sensitive, feasible, reliable, and cost-effective predictor to prevent and control cardiovascular disease and reduce mortality and disability.

## 2. Methods

### 2.1. Study population

A total of 701 patients who were admitted to the Second Hospital of Shandong University from January 2022 to December 2022 were included in the study. According to the patient's symptoms, signs and auxiliary examination results, they were divided into stable angina group, ACS group, and a control group without carotid and coronary artery disease (29–31). Exclusion criteria for the patients: (1) with severe liver and kidney dysfunction; (2) with severe arrhythmia, cardiomyopathy, valvular disease, and congenital heart disease; (3) with severe infection, thyroid function disease, blood system disease, autoimmune disease, tumor; (4) with systemic disease, severe diseases, and unstable vital signs; (5) recent severe trauma or major surgery; (6) pregnant or lactating women; (7) missing relevant important information.

Furthermore, a subset of 225 patients who underwent carotid ultrasound were selected from the above 701 patients. They were divided into low-risk plaque group, medium-to-high risk plaque group, and a control group without carotid plaques detected by ultrasound. Most importantly, the study was conducted in accordance with the ethical principles originated from the Declaration of Helsinki, and was also approved by the Ethical Committee of the Second Hospital of Shandong University.

### 2.2. General information of the subjects

General information of the subjects was collected in the study, including sex, age, blood pressure, heart rate, history of smoking, and past medical history (chronic diseases such as hypertension and diabetes). Morning fasting venous blood was collected and related laboratory indicators were inspected, including white blood cell (WBC), hemoglobin concentration (Hb), high-sensitivity C-reactive protein (CRP), fasting blood glucose (FBG), glycosylated hemoglobin (HbA1C), total cholesterol (TC), triglyceride (TG), low density lipoprotein cholesterol (LDL-C), high-density lipoprotein cholesterol (HDL-C), very low density lipoprotein cholesterol (VDLDL-C), small dense low-density lipoprotein cholesterol (SdLDL-C), cystatin C (Cys-C), homocysteine (HCY), uric acid (UA), brain natriuretic peptide (BNP), N-terminal pro B-type natriuretic peptide (NT-proBNP), troponin I (TnI), D-dimmer (D-D), aspartate aminotransferase (AST), alanine aminotransferase (ALT), estimated glomerular filtration rate (eGFR), and lipoprotein-associated phospholipase A2 (LP-PLA2). Within 24 h after admission, the left ventricular ejection fraction (LVEF) and carotid artery ultrasound were measured with cardiac color Doppler ultrasound (Sonosite M-Turbo, Bothell, WA, USA) to detect the presence, number, echogenicity, size, morphology, intima-media thickness (IMT), peak systolic velocity (PSV), and end-diastolic blood flow velocity (EDV) of left and/or right carotid artery plaques. The resistance index (RI) was calculated based on the relevant indicators [RI = (PSV-EDV) / PSV]. The information above was used to stratify the risk of carotid artery plaques.

### 2.3. Collection and storage of blood samples

All patients had their venous blood collected in the morning of the second day after admission. The blood was collected into a vacuum blood collection tube without anticoagulants. Prior to blood collection, each patient was informed of the study and had signed a consent form. After venous blood collection, the samples were sent to the Central Laboratory of the Second Hospital of Shandong University. The samples were allowed to clot for 20–30 min at 37°C, and then were centrifuged at 2000 xg for 10 min at 4°C. The upper serum layer was collected, avoiding the middle layer of blood cells and platelets. If hemolysis occurred during the collection process, the sample was discarded. Collected serum was stored in a 5 ml enzyme-free EP tube, and frozen at −80°C for later use, in a medical ultra-low temperature storage box.

### 2.4. Detection reagent

Primary antibodies for Western blotting analysis were purchased from Proteintech Group (Inc 5,400 Pearl Street, Suite 300 Rosemont, IL 60018, USA) as follows: TSG101 (28283-1-AP), CD81 (27855-1-AP). Goat Anti-Rabbit IgG (AS014) and Goat Anti-Mouse IgG (AS003) were from ABclonal Technology Co., Ltd. (Abclonal Technology, 86 Cummings Park Drive, Woburn, MA 01801, USA.). PVDF membranes were purchased from Millipore Corporation (St. Burlington, MA, USA.). ECL detection reagents (P0018S), SDS-PAGE gel configuration kit (P0012A), protein ladder (P0063), primary and secondary antibody dilution buffer (P0023A and P0023D), RIPA lysis buffer (P0013B) and Phosphate Buffered Saline (ST448-1l) were from Beyotime Biotechnology (Lane 1,500, Xinfei Road, Songjiang District, Shanghai, China). miRNA 1st strand cDNA synthesis kit (MR101-01) was from Vazyme (Kechuang Road, Nanjing, China). Tris buffered saline (G0001-2l), Tris-Glycine transfer buffer (G2017-1l), and Tris-Glycine SDS-PAGE running buffer (G2018-1l) were from Servicebio (No. 388, Gaoxin Second Road, Wuhan, China). Evo M-MLV RT Mix Kit with gDNA Clean for qPCR (AG11728) and SYBR Green Premix Pro Taq HS qPCR Kit (AG11701) were from Accurate Biology (No. 336, Shixue Road, Changsha, China). Tween-20 (IT9010) was from Solarbio Science & Technology (85A, Liandong U Valley, Tongzhou District, Beijing, China). TRIzol-LS reagent was from Thermo Fisher Scientific (5,781 Van Allen Way, Carlsbad, CA 92008, USA).

### 2.5. Exosome extraction and identification

Each subject fasted for 8–10 h before blood collection and collected blood samples with a non-anticoagulant vacuum tube (5 ml) before meal in the morning. After resting for 30 min, the serum samples were centrifuged by 2,000 xg/min for 15 min to remove impurities. Then the supernatant was centrifuged by 10,000 xg for 30 min to further remove impurities. The super serum was taken again and centrifuged by 120,000 xg for 70 min. The tube plate layer was precipitated, and some samples were colored. The main component of the precipitation was the exosomes. The precipitation was rinsed with phosphate buffer saline (PBS) solution and then centrifuged again at 120,000 xg for 70 min. The supernatant was carefully discarded and some adsorptive impurities were removed. The precipitation was the exosomes. After washing, the exosomes were suspended with 60 μl PBS. The morphology and particle size distribution of exosomes were observed by HT7800 (HITACHI, Japan) transmission electron microscopy (TEM). Nanoparticle tracking analysis (NTA) was performed using the ZetaView 8.04.02 SP2 system (ZetaView PMX110 machine, Particle Metrix, Germany) to detect the concentrations and size distribution of isolated exosomes. The exosomal labeled membrane proteins of TSG101 and CD81 were detected by Western blot. The identified extracellular vesicles in these extracellular precipitates were redissolved with PBS and frozen in a medical ultra-low temperature refrigerator at −80°C.

### 2.6. Extraction and detection of exosomal RNA

The Trizol-LS method was used to extract total RNA from the extracellular vesicles with the following steps: 1.5 ml nuclease-free EP tube was added into 750 μl Trizol-LS and 250 μl extracellular vesicle suspension and then cleavaged for 5 min at room temperature. Add 200*μ*l nucleic acid extraction solution (24:1) to the above solution, shake vigorously for 15 sec, and then store it on ice for 10 min to separate the phase. Place the separated mixture in a 4°C, 12,000 rpm centrifuge for 15 min. The supernatant was collected and transferred to another 1.5 ml nuclease-free EP tube. Isopropyl alcohol was added into the supernatant at a ratio of 1:1, mixed evenly, and RNA was precipitated overnight at −80°C. On the second day, remove the above mixture, thaw it, and centrifuge at 4°C and 12,000 rpm for 20 min. Remove the supernatant. Wash the RNA pellets three times with 75% ethanol without RNase, and on the last wash, aspirate all the liquid in the EP tube. Dry the precipitate in air. Add 20 μl DEPC water to dissolve air-dried precipitate and store at −80°C.

In the determination of ncRNAs concentration, after the frozen RNA was completely thawed, the RNA concentration was measured using a spectrophotometer, the concentration was recorded, and the circRNA and miRNA were reversely transcribed using a reverse transcription kit based on the RNA concentration. The expression of circSCMH1 and miR-874 was detected by qPCR. The primer sequence is shown in the following Supplementary Table S2. The circSCMH1 standard was diluted to different concentrations with a concentration gradient of 10, 1, 10^−1^, 10^−2^, 10^−3^, 10^−4^, 10^−5^, 10^−6^ (μM) and the miR-874 standard was diluted to a concentration gradient of 10^−1^, 10^−2^, 10^−3^, 10^−4^, 10^−5^, 10^−6^, 10^−7^ (μM). Draw a standard curve with the logarithm of each concentration as the X-axis and the corresponding CT value as the Y-axis. Based on the PCR results of each blood sample, the absolute concentrations of circSCMH1 and miR-874 and their ratios in each extracellular vesicle were calculated using a standard curve.

### 2.7. Statistical analysis

The experimental data was statistically analyzed and plotted using Graphpad Prism 5 and SPSS 26.0 software. Quantitative data that conformed to normal distribution were described using mean ± standard deviation, while quantitative data that did not conform to normal distribution were described using median + interquartile range. Qualitative data were described using composition ratio. Independent sample *t*-test or ANOVA was applied for quantitative data that conformed to normality and homogeneity of variance, and rank-sum test was used for other situations of quantitative data. Chi-square tests are used to compare rates between qualitative data. Logistic regression analysis was used to explore the correlation between different blood biochemical indicators and vital sign indicators with the occurrence of ACS, AMI, and high-risk plaques in the carotid artery. The lg (circSCMH1/miR-874) was evaluated whether it could be an independent risk factor for ACS and high-risk carotid plaques. ROC curve was used to detect the sensitivity and specificity of the above ratio prediction, and compared with the ratios proposed in previous studies, such as NLR, MLR, PLR, uric acid/neutrophil ratio, monocyte/HDL cholesterol ester ratio, triglyceride/HDL cholesterol ratio, etc., to evaluate its diagnostic value. For all analyses, *P* < 0.05 was considered statistically significant.

## 3. Results

### 3.1. Identification of Extracellular vesicles (EVs)

When observing EVs under transmission electron microscopy (TEM), it can be seen that there are circular bilayer membrane vesicles with a diameter of 30–150 nm, typically appearing as biconcave discs (Supplementary Figures S1A–C), preliminarily indicating successful extraction of EVs. The particle size of the EVs was analyzed and detected using a nanoparticle size analyzer, and the results were shown in Supplementary Figure S1D. Based on NTA test results, there were no significant differences in exosomes concentrations between the three groups. The identification results showed that the particle size was consistent with the size of exosomes. Western blot was used to detect the expression levels of two protein markers on the surface of EVs (TSG101 and CD81, with molecular weights of 46 kDa and 22 kDa, respectively), and the results were shown in Supplementary Figures S1E,F. The results demonstrated that the labeled proteins TSG101 and CD81 extracted from the exosomes were positive (Supplementary Figures S1E,F).

### 3.2. Statistical analysis of circSCMH1 to miR-874 ratio and coronary plaque stability

#### 3.2.1. Baseline characteristics

**Figure 1:**
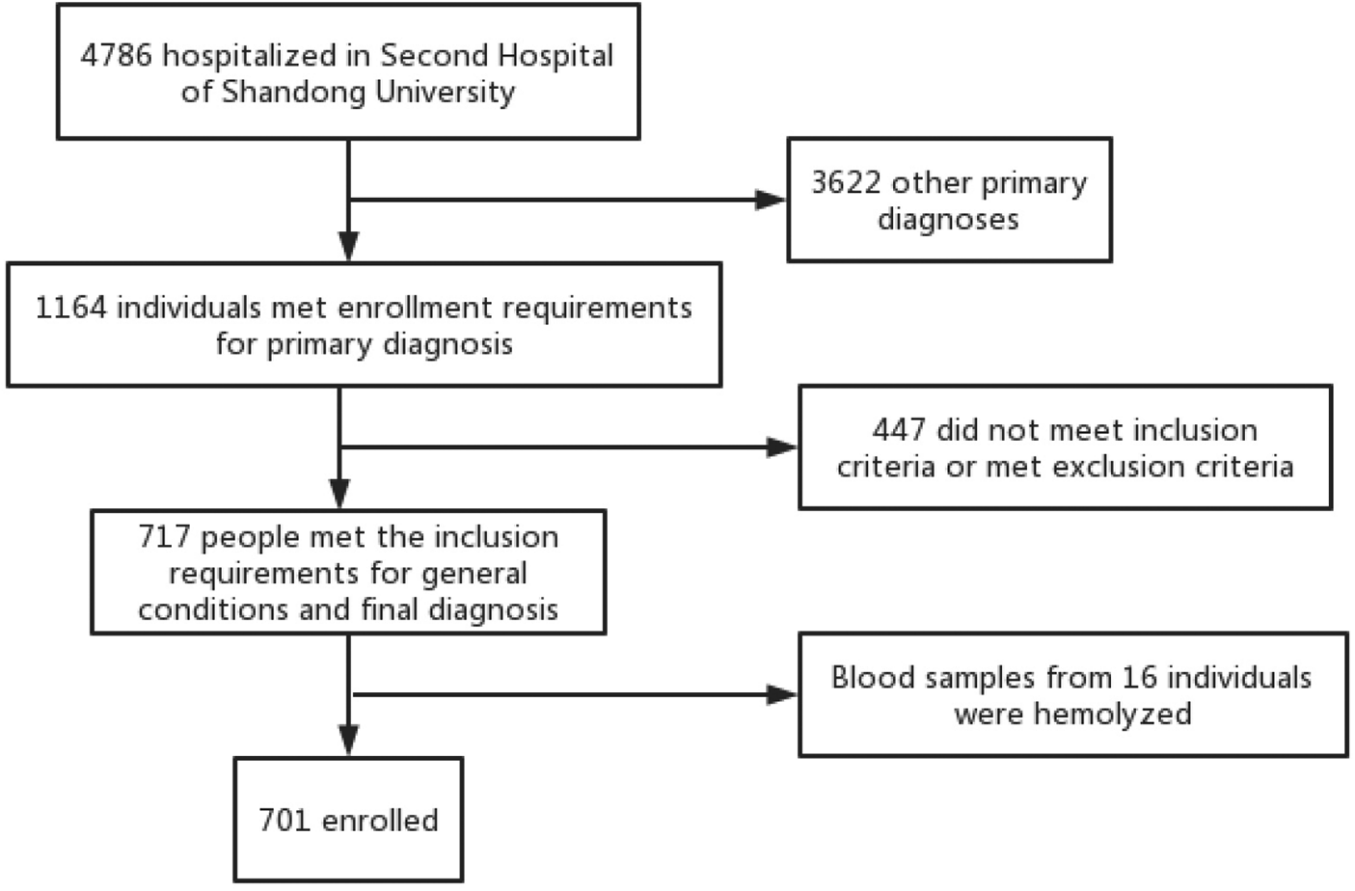
Flowchart of the study.

A total of 701 patients who were admitted to the Second Hospital of Shandong University from January 2022 to December 2022 were included in the study (Figure 1). The comparison of general information among the three groups is shown in Table 1. The sex composition of the data indicated 370 males and 331 females (Table 1). The oldest was 69 years old, the youngest was 41 years old, and the average age was 58.19 ± 7.40 years. All patients were divided into 3 groups: control group (101 cases), ACS group (300 cases), and stable coronary artery disease group (300 cases). There were no statistically significant differences in the HR, Hb, PLT, VLDL-C, HCY, HbA1c, SdLDL-C, and AST levels among the three groups (*P* > 0.05). Compared with the control group, the stable coronary artery disease group and the ACS group had larger age, higher proportion of hypertension (HPT), diabetes, and smoking histories, higher systolic blood pressure (SBP), diastolic blood pressure (DBP), pulse pressure difference (PPD), and mean arterial pressure (MAP) (all *P* < 0.05) (Table 1). The WBC, cys-C, UA, TC, triglycerides, LDL-C, D-D and BNP levels in the SCAD and the ACS groups were also higher than those of the control group. Meanwhile, the eGFR, HDL-C and LVEF levels were lower in the patients with SCAD and ACS (all *P* < 0.05). Furthermore, the SCAD group had higher LP-PLA2 (*P* < 0.05), while the ACS group had no significant difference in LP-PLA2 compared with the control group (*P* > 0.05). When comparing the ACS group with the SCAD group, there were no statistically significant differences in age, SBP, DBP, PPD, MAP, TC, triglycerides, LDL-C, and LP-PLA2 levels (*P* > 0.05). But compared with SCAD group, the proportion of male patients, hypertension, diabetes, smoking history, FBG, WBC, Cys-C, D-D, BNP, NT-proBNP, ALT, and UA levels were higher, while the eGFR, HDL-C, and LVEF were lower in patients with ACS (all *P* < 0.05) (Table 1). Previous studies have shown that components of metabolic syndrome such as diabetes, hypertension, and hyperlipidemia are strongly associated with coronary atherosclerotic lesions (16). However, some of the results of this study are inconsistent with the traditional view, which may be related to the use of hypoglycemic, hypertensive and lipid-lowering drugs before their admission, which may affect the true levels of blood pressure, blood glucose and lipids of these patients.

**Table 1:** Baseline characteristics of patients in ACS, SCAD and control groups.

Project	ACS (*n* = 300)	SCAD (*n* = 300)	Control (*n* = 101)	*P*		
				*P1*	*P2*	*P3*
Age (year)	62.95 ± 11.807	64.12 ± 10.905	50.80 ± 12.402	<0.001	<0.001	0.208
Male sex [case(%)]	206 (68.7%)	125 (41.7%)	39 (38.6%)	<0.001^a^		
SBP (mmHg)	157.27 ± 26.739	158.81 ± 28.622	126.45 ± 16.315	<0.001	<0.001	0.501
DBP (mmHg)	95.99 ± 21.289	95.82 ± 19.325	90.34 ± 14.696	0.014	0.003	0.920
PPD (mmHg)	56.40 ± 22.385	60.53 ± 20.778	54.70 ± 17.080	0.427	0.005	0.018
MAP (mmHg)	115.09 ± 21.268	116.06 ± 21.334	108.57 ± 17.966	0.006	0.001	0.579
HR (bpm)	77.09 ± 11.107	74.50 ± 10.671	75.30 ± 11.710	0.168	0.529	0.054
Smoke [case(%)]	126 (42.0%)	89 (29.7%)	21 (20.8%)	<0.001^b^		
Hypertension [case(%)]	184 (61.3%)	195 (65.0%)	3 (3.0%)	<0.001^b^		
Diabetes [case(%)]	91 (30.3%)	83 (27.7%)	2 (2.0%)	<0.001^b^		
WBC (×10^9^/L)	7.55 ± 2.851	6.19 ± 1.729	5.66 ± 1.589	<0.001	0.007	<0.001
Hb (g/L)	137.95 ± 24.672	136.70 ± 24.784	137.37 ± 16.378	0.829	0.802	0.543
PLT (×10^12^/L)	229.96 ± 60.538	223.73 ± 47.391	234.22 ± 53.533	0.534	0.066	0.167
FBG (mmol/L)	6.54 ± 2.848	6.13 ± 1.927	6.12 ± 2.248	0.191	0.966	0.044
Cys-C (mg/L)	1.14 ± 0.270	1.09 ± 0.232	0.99 ± 0.133	<0.001	<0.001	0.023
eGFR [ml/(min*m^2^)]	93.69 ± 23.434	98.72 ± 24.821	108.32 ± 22.381	<0.001	0.001	0.012
UA (μmol/L)	330.72 ± 89.204	315.55 ± 83.389	289.13 ± 76.520	<0.001	0.006	0.034
TC (mmol/L)	4.36 ± 1.131	4.34 ± 1.168	4.86 ± 1.354	0.001	<0.001	0.805
TG (mmol/L)	1.29 (0.94,1.85)	1.21 (0.93,1.87)	1.11 (0.75,1.53)	0.002	0.013	0.384
LDL-C (mmol/L)	2.56 ± 0.919	2.50 ± 0.952	2.97 ± 0.962	<0.001	<0.001	0.407
HDL-C (mmol/L)	1.10 ± 0.314	1.21 ± 0.324	1.30 ± 0.311	<0.001	0.018	<0.001
VLDL-C (mmol/L)	0.31 ± 0.216	0.29 ± 0.170	0.31 ± 0.278	0.976	0.423	0.259
SdLDL-C (mmol/L)	0.73 ± 0.34	0.73 ± 0.322	0.81 ± 0.405	0.117	0.140	0.871
ALT (U/L)	23.89 ± 20.958	20.43 ± 14.239	21.83 ± 18.076	0.391	0.441	0.021
AST (U/L)	33.70 ± 49.695	27.17 ± 39.870	31.52 ± 54.375	0.717	0.406	0.084
HCY (μmol/L)	16.05 ± 11.676	15.59 ± 9.332	15.72 ± 9.038	0.819	0.910	0.637
HbA1c (NGSP%)	6.69 ± 1.504	6.48 ± 1.193	6.63 ± 1.529	0.796	0.493	0.157
HbA1c (IFCC%)	49.57 ± 16.437	47.36 ± 13.037	48.95 ± 16.708	0.798	0.493	0.158
LP-PLA2 (IU/L)	474.69 ± 131.374	461.38 ± 122.594	497.11 ± 126.680	0.216	0.043	0.283
CRP (mg/L)	1.70 (1.000,5.400)	1.00 (1.000,2.50)	1.30 (1.000,2.725)	0.102	0.924	0.054
TnI (ng/ml)	0.011 (0.009,1.566)	0.010 (0.004,0.017)	0.010 (0.003,0.019)	0.002	0.711	<0.001
D-D (μg/m)	0.38 (0.26,0.63)	0.32 (0.21,0.51)	0.27 (0.18,0.38)	<0.001	0.012	0.001
BNP (pg/m)	68.05 (30.00,154.53)	41.55 (23.23,81.30)	24.00 (14.80,42.40)	<0.001	<0.001	<0.001
NT pro-BNP (pg/ml)	326.30 (85.85,750.55)	82.50 (30.33,155.50)	–	–	–	<0.001
LVEF (%)	58.31 ± 7.699	63.22 ± 4.162	64.72 ± 3.185	<0.001	0.006	<0.001

^a^ There was significant difference between the ACS and control group, and the ACS group and SCAD group, but no between the SCAD group and control group.

^b^ There was significant difference between the ACS and control group, and the control group and SCAD group, but no between the SCAD group and ACS group.

*P1*, ACS versus control; *P2*, SCAD vs. control; *P3,* ACS vs. SCAD; Comparition of Male sex, Smoke, Hypertension, Diabetes between groups used the χ^2^ test; Comparition of TG, CRP, TnI, D-D, BNP, NT-proBNP between groups used the Rank sum test, and other projects’ comparition used t test; ACS, acute coronary syndrome; SCAD, stable coronary artery disease; SBP, systolic blood pressure; DBP, diastolic blood pressure; PPD, Pulse pressure difference; MAP, mean arterial pressure; HR, heart rate; WBC, white blood cell; Hb, hemoglobin; PLT, Platelet; FBG, fasting blood glucose; Cys-C, Cystatin C; eGFR, estimated glomerular filtration rate; UA, uric acid; TC, total cholesterol; TG, triglycerides; LDL-C, low density lipoprotein cholesterol; HDL-C, high density lipoprotein cholesterol; VLDL-C, very low density lipoprotein cholesterol; SdLDL-C, small and low density lipoprotein cholesterol; AST, aspartate aminotransferase; ALT, alanine aminotransferase; HCY, homocysteine; HbA1c, glycosylated hemoglobin A1c; LP-PLA2, lipoprotein-associated phospholipase 2; CRP, C-reactive protein; TnI, troponin I; D-D, D-dimer; BNP, B-type brain natriuretic peptide; NT-proBNP, N-terminal pro-B-type natriuretic peptide; LVEF, left ventricular ejection fraction.

#### 3.2.2. Relationship between circSCMH1/miR-874 ratio and coronary artery disease

**Figure 2:**
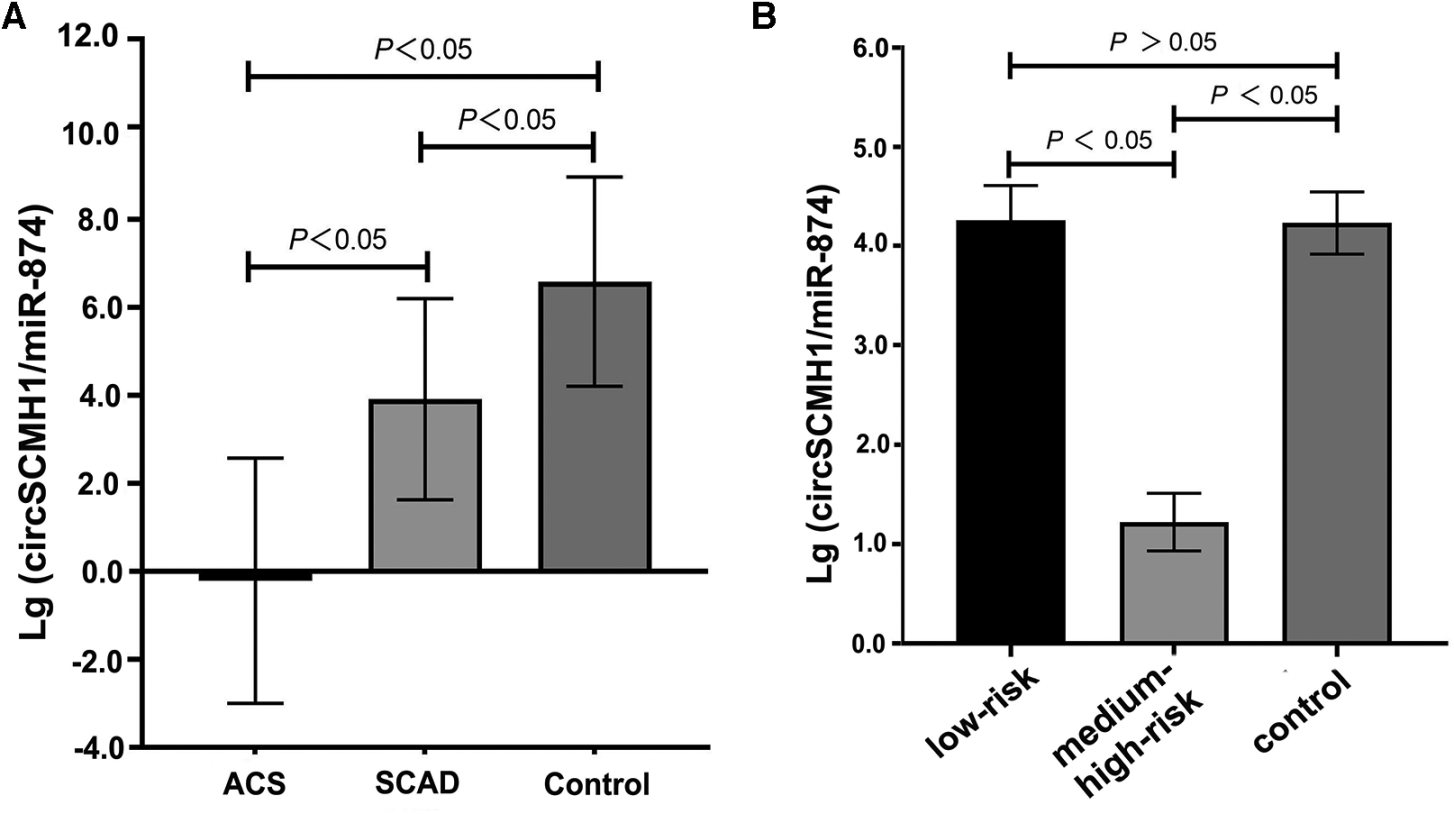
Comparison of lg (circSCMH1/miR-874). (**A**) comparison among ACS, SCAD and control patients; (**B**) comparison among low-risk plaque, medium-high risk plaque and control groups. Error bars represent standard deviation. ANOVA test was performed between the three groups.

The ratio of circSCMH1 to miR-874 in peripheral blood was logarithmized, and then lg (circSCMH1/miR-874) was compared among the SCAD, ACS, and control groups. Compared with the SCAD and control groups, the ACS group showed a decrease in lg (circSCMH1/miR-874) (*P* < 0.05) (Figure 2A). Compared with the control group, the SCAD group also showed a decrease in lg (circSCMH1/miR-874) (*P* < 0.05) (Figure 2A). Details of the comparison between the typical CCTA images (Supplementary Figure S2) and lg (circSCMH1/ miR-874) (Figure 2A) among the three groups were shown in Supplementary Figure S2 and Figure 2A. It is not difficult to see that the circSCMH1/miR-874 ratio presents a significant negative correlation with coronary artery disease, that is, with the reduction of circSCMH1/miR-874 ratio, the degree of coronary artery disease becomes more serious (Figure 2A, Supplementary Figure S2).

#### 3.2.3. Association between circSCMH1/miR-874 ratio and AMI /ACS

**Figure 3:**
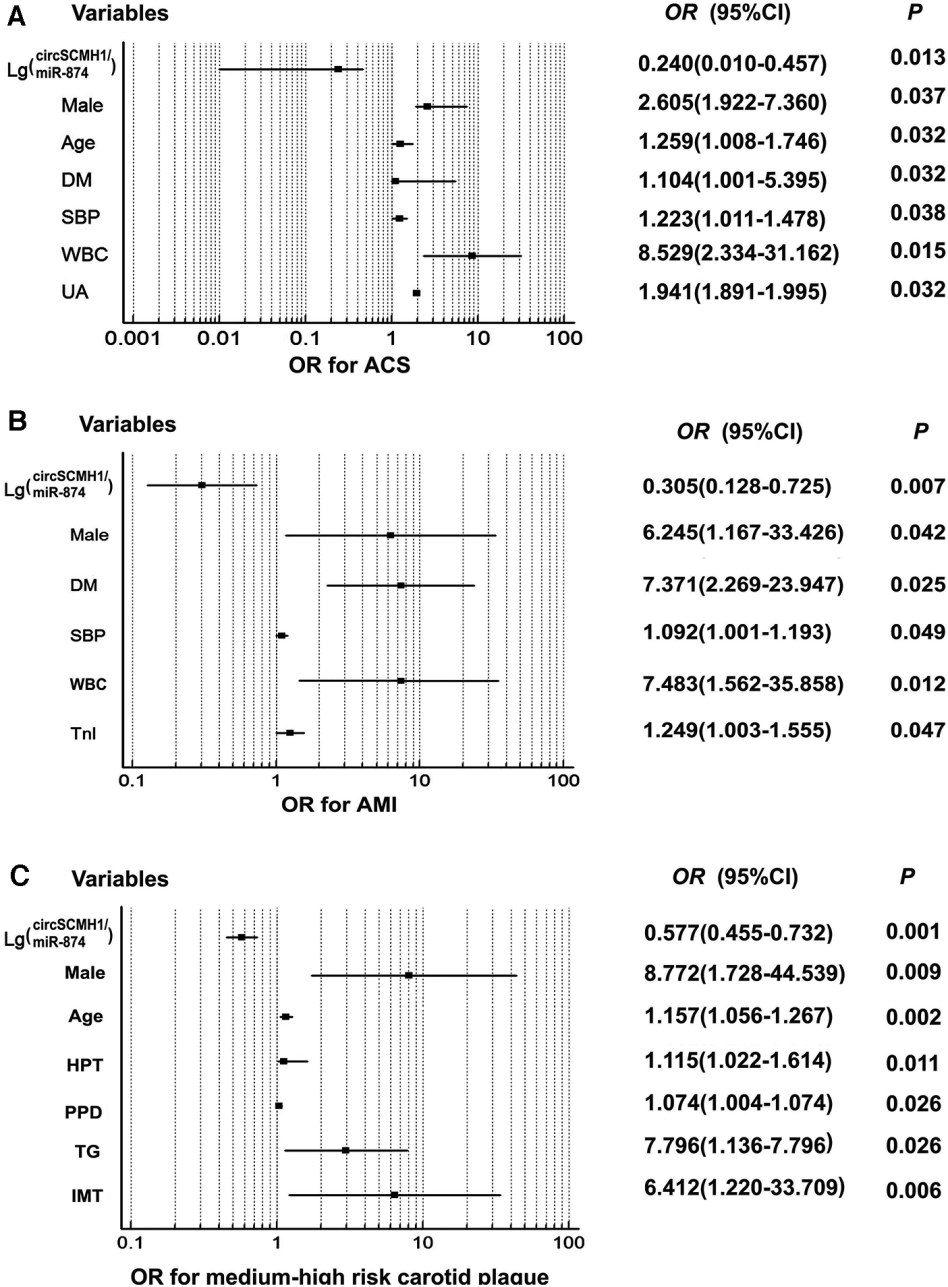
Forest plot demonstrated clinical risk factors for ACS, AMI, and medium-high risk carotid plaque. Forest plot demonstrated clinical risk factors for ACS, AMI, and medium-high risk carotid plaque; (**A**) risk factors for ACS; (**B**) risk factors for AMI; (**C**) risk factors for medium-high risk carotid plaque. DM, diabetes mellitus; SBP, systolic blood pressure; WBC, white blood cell; UA, uric acid; Tn I, troponin I; PPD, pulse pressure difference.

Univariate and multivariate logistic regression analyses were performed using age, sex, past medical history, hemodynamics, clinical biochemistry and other indicators including lg (circSCMH1/miR-874), and whether the patient was diagnosed with ACS or whether the patient experienced AMI as dependent variables. Consequently, when the dependent variable was whether the patient was diagnosed with ACS, the univariate logistic regression analysis demonstrated that male, age, history of smoking, hypertension and type 2 diabetes, SBP, WBC, FBG, Cys-C, UA, HDL-C, D-D, BNP, TnI, LVEF, ALT, and lg (circSCMH1/miR-874) were statistically associated with ACS occurrence. Furthermore, multivariate logistic regression analysis, after adjusting for potential confounding variables, showed that male, age, SBP, history of type 2 diabetes, WBC, Cys-C, UA, and lg(circSCMH1/miR-874) were independent risk factors for ACS. Among them, the risk of ACS increased with the decrease of lg (circSCMH1/miR-874) (all *P* < 0.05) (Table 2, Figure 3A). On the other hand, using whether the patient has experienced AMI as the dependent variable, the univariate logistic regression analysis demonstrated that male, age, the history of smoking and type 2 diabetes, WBC, UA, TC, LDL-C, HDL-C, ALT, LP-PLA2, D-D, TnI, BNP, lg(circSCMH1/miR-874), and LVEF were statistically associated with AMI attack. Further multivariate logistic regression analysis after controlling confounding variables showed that male, type 2 diabetes history, SBP, TnI, WBC, and lg(circSCMH1/miR-874) were independent risk factors for AMI, of which, the risk of AMI outbreak increased with the decrease of lg(circSCMH1/miR-874) (*P* < 0.05) (Table 3, Figure 3B).

**Table 2:** Logistic regression analysis stratified by ACS **.**

Project	Univariate Logistic Regression			Multivariate Logistic Regression		
	OR	95% CI	*P*	OR	95% CI	*P*
Lg (circSCMH1/ miR-874)	0.563	0.520–0.610	<0.001	0.240	0.001–0.457	0.013
Male sex [case(%)]	3.167	2.312–4.339	<0.001	2.605	1.922–7.360	0.037
Age (year)	1.015	1.002–1.027	0.021	1.259	1.008–1.746	0.032
Smoke [case(%)]	1.892	1.378–2.597	<0.001			
Hypertension [case(%)]	1.626	1.200–2.204	0.002	–	–	–
Diabetes [case(%)]	1.619	1.148–2.282	0.006	1.104	1.001–5.395	0.032
SBP (mmHg)	1.008	1.003–1.013	0.003	1.223	1.011–1.478	0.038
DBP (mmHg)	1.004	0.996–1.012	0.305	–	–	–
PPD (mmHg)	0.994	0.987–1.001	0.096	–	–	–
MAP (mmHg)	1.002	0.995–1.009	0.567	–	–	–
WBC (×10^9^/L)	1.367	1.261–1.482	<0.001	8.529	2.334–31.162	0.015
FBG (mmol/L)	1.074	1.006–1.146	0.031	–	–	–
Cys-C (mg/L)	3.816	1.841–7.909	<0.001	–	–	–
eGFR [ml/(min*m^2^)]	0.987	0.980–0.994	<0.001	–	–	–
UA (μmol/L)	1.003	1.001–1.005	0.001	1.941	1.891–1.995	0.032
TC (mmol/L)	0.925	0.810–1.057	0.253	–	–	–
TG (mmol/L)	1.116	0.960–1.298	0.152	–	–	–
LDL-C (mmol/L)	0.952	0.808–1.121	0.552	–	–	–
HDL-C (mmol/L)	0.258	0.145–0.460	<0.001	–	–	–
ALT (U/L)	1.010	1.001–1.019	0.032	–	–	–
TnI (ng/ml)	1.036	1.015–1.058	0.001	–	–	–
LP-PLA2 (IU/L)	1.000	0.999–1.002	0.696	–	–	–
D-D (μg/ml)	2.010	1.301–3.108	0.002	–	–	–
LVEF (%)	0.828	0.794–0.863	<0.001	–	–	–
BNP (pg/ml)	1.032	1.005–1.059	0.019	–	–	–

ACS, acute coronary syndrome; SCAD, stable coronary artery disease; CI, confidence interval; SBP, systolic blood pressure; DBP, diastolic blood pressure; PPD, Pulse pressure difference; MAP, mean arterial pressure; WBC, white blood cell; FBG, fasting blood glucose; Cys-C, Cystatin C; eGFR, estimated glomerular filtration rate; UA, uric acid; TC, total cholesterol; TG, triglycerides; LDL-C, low density lipoprotein cholesterol; HDL-C, high density lipoprotein cholesterol; ALT, alanine aminotransferase; TnI, troponin I; LP-PLA2, lipoprotein-associated phospholipase 2; D-D, D-dimer; BNP, B-type brain natriuretic peptide; LVEF, left ventricular ejection fraction.

**Table 3:** Logistic regression analysis classified by AMI**.**

Project	Univariate Logistic Regression			Multivariate Logistic Regression		
	OR	95% CI	*P*	OR	95% CI	*P*
Lg (circSCMH1/ miR-874)	0.644	0.595–0.696	<0.001	0.305	0.128–0.725	0.007
Male sex [case(%)]	3.118	2.147–4.530	<0.001	6.245	1.167–33.426	0.042
Age (year)	1.000	0.987–1.014	0.971	–	–	–
Smoke [case(%)]	1.718	1.209–2.441	0.003	–	–	–
Hypertension [case(%)]	1.206	0.854–1.703	0.287	–	–	–
Diabetes [case(%)]	1.518	1.040–2.215	0.031	7.371	2.269–23.947	0.025
SBP (mmHg)	1.005	0.999–1.011	0.134	1.092	1.001–1.193	0.049
DBP (mmHg)	1.005	0.997–1.014	0.220	–	–	–
PPD (mmHg)	0.994	0.986–1.002	0.158	–	–	–
MAP (mmHg)	1.003	0.994–1.011	0.530	–	–	–
WBC (×10^9^/L)	1.528	1.396–1.671	<0.001	7.483	1.562–35.858	0.012
FBG (mmol/L)	1.046	0.977–1.121	0.194	–	–	–
Cys-C (mg/L)	1.873	0.899–3.905	0.094	–	–	–
eGFR [ml/(min*m^2^)]	0.994	0.986–1.001	0.082	–	–	–
UA (μmol/L)	1.003	1.001–1.005	0.002	–	–	–
TC (mmol/L)	1.182	1.018–1.373	0.028	–	–	–
TG (mmol/L)	1.074	0.917–1.259	0.376	–	–	–
LDL-C (mmol/L)	1.388	1.151–1.674	0.001	–	–	–
HDL-C (mmol/L)	0.341	0.175–0.663	0.002	–	–	–
TnI (ng/ml)	1.049	1.028–1.071	<0.001	1.249	1.003–1.555	0.047
ALT (U/L)	1.012	1.003–1.022	0.007	–	–	–
LP-PLA2 (IU/L)	1.003	1.001–1.005	<0.001	–	–	–
D-D (μg/ml)	3.150	1.923–5.161	<0.001	–	–	–
LVEF (%)	0.771	0.732–0.813	<0.001	–	–	–
BNP (pg/ml)	1.004	1.002–1.006	<0.001	–	–	–

ACS, acute coronary syndrome; SCAD, stable coronary artery disease; CI, confidence interval; AMI, acute myocardial infarction; SBP, systolic blood pressure; DBP, diastolic blood pressure; PPD, Pulse pressure difference; MAP, mean arterial pressure; WBC, white blood cell; FBG, fasting blood glucose; Cys-C, Cystatin C; eGFR, estimated glomerular filtration rate; UA, uric acid; TC, total cholesterol; TG, triglycerides; LDL-C, low density lipoprotein cholesterol; HDL-C, high density lipoprotein cholesterol; ALT, alanine aminotransferase; TnI, troponin I; LP-PLA2, lipoprotein-associated phospholipase 2; D-D, D-dimer; BNP, B-type brain natriuretic peptide; LVEF, left ventricular ejection fraction.

#### 3.2.4. Comparison of predictive value of related different ratios for ACS and AMI

ROC curve analysis was conducted using NLR, MLR, PLR, TG/HDL-C ratio, monocyte/ HDL-C ratio, and uric acid/neutrophil ratio, and lg (circSCMH1/miR-874) as independent variables to compare their diagnostic values for ACS or AMI of subjects.

**Figure 4:**
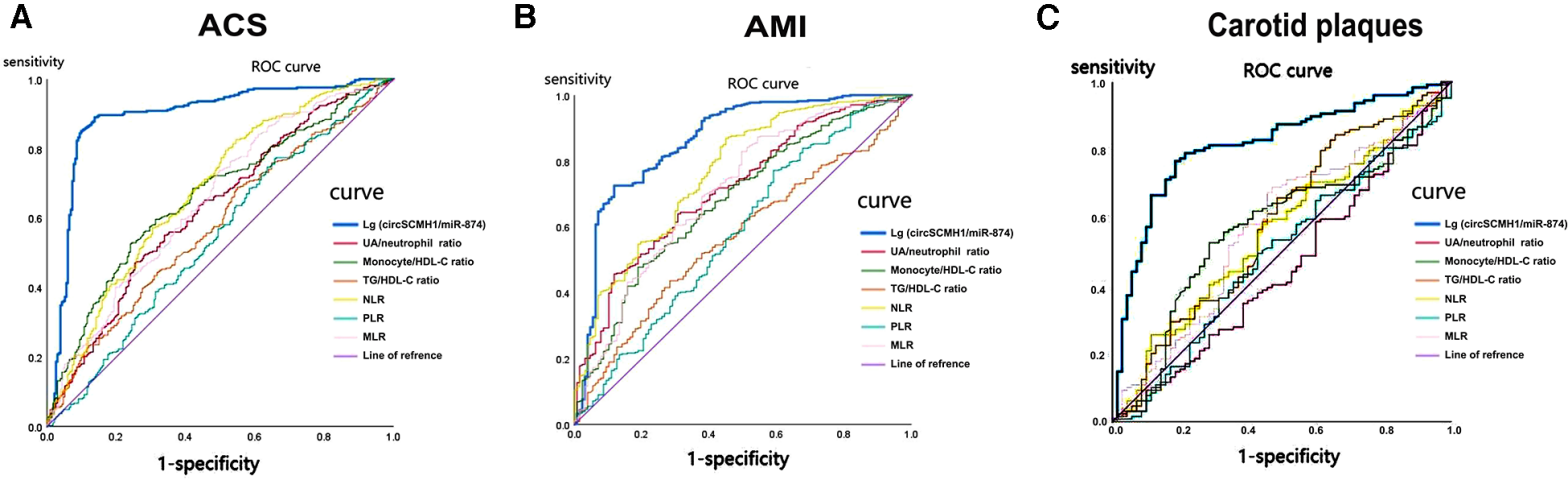
The ROC curve of lg (circSCMH1/miR-874) for ACS, AMI and medium-high risk plaque. (**A**) ROC curve of lg (circSCMH1/miR-87) for ACS; (**B**) ROC curve of lg (circSCMH1/miR-874) for AMI; (**C**) ROC curve of lg (circSCMH1/miR-874) predicting medium-high-risk plaque. NLR, neutrophil/lymphocyte; PLR, platelet/lymphocyte; MLR, monocyte/lymphocyte.

With regard to the diagnosis of ACS (Table 4, Figure 4A), the ROC curve analysis demonstrated that the area under the curve (AUC) of lg (circSCMH1/miR-874) was 0.893 (95% CI: 0.862–0.923), with a sensitivity of 89.0%, a specificity of 86.3%, and a cut-off value of 1.52. The AUC for uric acid/neutrophil ratio was 0.628 (95% CI: 0.581–0.675), with a sensitivity of 47.1%, a specificity of 74.7%, a cut-off value of 95.51. The AUC for monocyte/HDL-C ratio was 0.662 (95% CI: 0.617–0.707), with a sensitivity of 52.6%, a specificity of 76.0%, and a cut-off value of 0.36. The AUC for TG/HDL-C ratio was 0.577 (95% CI: 0.530–0.625), with a sensitivity of 68.8%, a specificity of 45.1%, and a cut-off value of 1.33. The AUC for NLR was 0.680 (95% CI: 0.635–0.725), with a sensitivity of 82.6%, a specificity of 45.5%, and a cut-off value of 2.86. The AUC for PLR was 0.544 (95% CI: 0.495–0.593), with a sensitivity of 77.1%, a specificity of 34.3%, and a cut-off value of 161.29. The AUC for MLR was 0.651 (95% CI: 0.605–0.698), with a sensitivity of 73.7%, a specificity of 50.2%, and a cut-off value of 1.54 (Table 4).

**Table 4:** Comparison of ROC curves for different ratios in predicting ACS.

Project	AUC	sensitivity	specificity	cut-off value	95% CI
Lg (circSCMH1/miR-874)	0.893	89.0%	86.3%	1.52	0.862–0.923
UA/neutrpphil ratio	0.628	47.1%	74.7%	95.51	0.581–0.675
monocyte/HDL-C ratio	0.662	52.6%	76.0%	0.36	0.617–0.707
TG/HDL-C ratio	0.577	68.8%	45.1%	1.33	0.530–0.625
NLR	0.680	82.6%	45.5%	2.86	0.635–0.725
PLR	0.544	77.1%	34.3%	161.29	0.495–0.593
MLR	0.651	73.7%	50.2%	1.54	0.605–0.698

AUC, area under curve; CI, confidence interval; UA, uric acid; NLR, neutrophil/lymphocyte ratio; PLR, platelet/lymphocyte ratio; MLR, monocyte/lymphocyte ratio.

In terms of AMI diagnosis (Table 5, Figure 4B), the AUC of lg (circSCMH1/miR-874) was 0.862 (95% CI: 0.822–0.902), with a sensitivity of 72.5%, a specificity of 88.2%, and a cut-off value of 1.38. The AUC of uric acid/neutrophil ratio was 0.716 (95% CI: 0.667–0.765), with a sensitivity of 45.7%, a specificity of 88.2%, and a cut-off value of 95.51. The AUC of monocyte/HDL-C ratio was 0.679 (95% CI: 0.627–0.731), with a sensitivity of 47.8%, a specificity of 81.1%, and a cut-off value of 0.36. The AUC of TG/HDL-C ratio was 0.568 (95% CI: 0.514–0.621), with a sensitivity of 43.6%, a specificity of 71.7%, and a cut-off value of 0.93. The AUC of NLR was 0.765 (95% CI: 0.718–0.813), with a sensitivity of 86.8%, a specificity of 55.1%, and a cut-off value of 3.13. The AUC of PLR was 0.583 (95% CI: 0.524–0.642), with a sensitivity of 77.1%, a specificity of 40.9%, and a cut-off value of 161.29. The AUC of the ROC curve for MLR was 0.706 (95% CI: 0.652–0.759), with a sensitivity of 82.9% and a specificity of 50.4%, and a cut-off value of 0.35 (Table 5, Figure 4B). As could be seen from the above, lg (circSCMH1/miR-874) had a good predictive value for ACS and AMI, and its comprehensive predictive value was better than that of other previously predicted ratios such as NLR, MLR, PLR, uric acid/neutrophil, monocyte/HDL-C, and triglyceride/HDL-C ratios.

**Table 5:** Comparison of ROC curves for different ratios in predicting AMI.

Project	AUC	Sensitivity	Specificity	Cut-off value	95% CI
Lg (circSCMH1/hsa-miR-874)	0.862	72.5%	88.2%	1.38	0.822–0.902
UA/neutrpphil ratio	0.716	45.7%	88.2%	95.51	0.667–0.765
monocyte/HDL-C ratio	0.679	47.8%	81.1%	0.36	0.627–0.731
TG/HDL-C ratio	0.568	43.6%	71.7%	0.93	0.514–0.621
NLR	0.765	86.8%	55.1%	3.13	0.718–0.813
PLR	0.583	77.1%	40.9%	161.29	0.524–0.642
MLR	0.706	82.9%	50.4%	0.35	0.652–0.759

AUC, area under curve; CI, confidence interval; UA, uric acid; NLR, neutrophil/lymphocyte; PLR, platelet/lymphocyte; MLR, monocyte/lymphocyte.

### 3.3. Correlation between circSCMH1/miR-874 ratio and carotid plaque stability

A total of 225 patients were included in the study, including 158 patients with carotid plaque, 67 patients without carotid plaque, detected by ultrasound, respectively. The research subjects included 114 males and 111 females. The oldest was 69 years old, the youngest was 41 years old, and the average age was 63.32 ± 9.74 years. This study adopted “Ultrasound Evaluation of Carotid Plaque Risk Level” jointly proposed by the Beijing Anzhen Hospital Ultrasound Department and Neurology Department in 2016, and the risk level of carotid plaque was assessed with the carotid ultrasound results. The 225 patients were divided into low-risk plaque group (73 cases), medium-high risk plaque group (85 cases), and control group (67 cases), with the control group being hospitalized patients who underwent carotid ultrasound but did not have carotid plaque detected. The brief standard for carotid risk level assessment is as follows: Low-risk plaque: (1) small homogenous low echogenic plaque (flat plaque) or low echogenicity mixed plaque with plaque thickness <2 mm; (2) homogenous hyperechoic plaque or mainly hyperechoic mixed plaque. Medium-risk plaque: homogenous low echogenic plaque or mixed plaque with low echogenicity as the main feature with plaque thickness of 2–3 mm. High-risk plaque: (1) ulcerative plaque; (2) homogenous low echogenic plaque or mixed plaque with low echogenicity as the main feature, with thickness greater than 3 mm and length greater than 15 mm; (3) heterogeneous low echogenic plaque or mixed plaque with low echogenicity as the main feature with a lipid necrotic core; (4) plaque formation with neovascularization. Very high-risk plaque: combined with high-risk plaque, (1) plaque with fibrous cap rupture; (2) plaque with attached active small thrombus; (3) plaque with ulcerative surface accompanied by active small thrombus formation; (4) active ulcerative plaque; (5) plaque with attached active large thrombus; (6) jellyfish plaque; (7) Plaque with ulcerative surface accompanied by active large thrombus formation.

#### 3.3.1. Clinical data characteristics for stability analysis of carotid artery plaques

There were no statistically significant differences in the HR, SBP, MAP, WBC, Hb, TG, TC, LDL-C, HDL-C, LP-PLA2, D-D, BNP, TnI, HbA1c, HCY, FBG, VLDL-C, SdLDL-C, AST, ALT, CRP or other indicators among the low-risk plaque group, medium-high-risk plaque group, and control group (all *P *> 0.05) (Table 6). Compared with the control group, the IMT was thicker in both the low-risk plaque and the medium-high-risk plaque groups. The medium-high-risk plaque group had older age, more males, higher SBP, a greater proportion of patients with a history of hypertension, diabetes, and smoking. And patients in the low-risk plaque and the medium-high risk plaque groups had higher levels of Cys-C, UA, and RI, and lower levels of eGFR, PLT, and EDV, than those of control group. The difference in pulse pressure was not statistically significant (*P *> 0.05) among these groups (Table 6). Furthermore, there were no statistically significant differences in sex, the history of diabetes and smoking in the low-risk plaque group compared with the control group (*P *> 0.05). There were no statistically significant differences in SBP, IMT, and the history of diabetes, smoking, and hypertension between the low-risk plaque and the medium-high risk plaque groups (*P *> 0.05) (Table 6). However, the medium-high risk plaque group had older age, more males, higher PPD, RI, and TG levels, and lower VLDL-C levels (Table 6). The differences in FBG, blood lipid, and other indicators among the three groups were not consistent with previous studies, which may be related to the use of hypoglycemic and lipid-lowering drugs by the enrolled patients before enrollment, which might have affected the levels of FBG and blood lipids measured after enrollment (Table 6).

**Table 6:** Baseline characteristics of patients in carotid plaque and control groups.

Project	Low (*n* = 73)	Medium-high (*n* = 85)	Control (*n* = 67)	*P*		
				*P1*	*P2*	*P3*
Age (year)	64.23 ± 10.405	68.01 ± 8.686	59.23 ± 12.437	0.008	<0.001	0.015
Male sex [case(%)]	29 (39.7%)	54 (63.5%)	31 (39.2%)	0.002^a^		
SBP (mmHg)	156.40 ± 26.958	163.99 ± 26.530	146.29 ± 29.098	0.028	<0.001	0.080
DBP (mmHg)	97.45 ± 20.783	92.57 ± 19.310	91.84 ± 16.894	0.069	0.798	0.130
PPD (mmHg)	57.60 ± 20.607	64.82 ± 23.120	59.47 ± 18.290	0.555	0.101	0.041
MAP (mmHg)	116.65 ± 21.816	114.69 ± 21.528	111.66 ± 20.596	0.149	0.361	0.574
HR (bpm)	74.52 ± 9.418	74.50 ± 10.671	73.09 ± 9.879	0.755	0.537	0.357
Smoke [case (%)]	21 (28.8%)	37 (43.5%)	18 (22.8%)	0.013^a^		
Hypertension[case (%)]	39 (53.4%)	62 (72.9%)	35 (44.3%)	0.001^b^		
Diabetes [case (%)]	16 (21.9%)	30 (35.3%)	14 (17.7%)	0.026^a^		
WBC (×10^9^/L)	6.60 ± 1.712	6.33 ± 1.560	6.32 ± 2.074	0.379	0.974	0.312
Hb (g/L)	136.33 ± 13.713	137.91 ± 26.909	138.12 ± 36.097	0.690	0.968	0.653
PLT (×10^12^/L)	229.18 ± 40.230	215.74 ± 56.060	237.28 ± 60.084	0.337	0.021	0.087
FBG (mmol/L)	5.99 ± 1.996	6.58 ± 3.332	6.44 ± 2.440	0.225	0.773	0.196
Cys-C (mg/L)	1.11 ± 0.176	1.17 ± 0.208	1.04 ± 0.246	0.059	0.002	0.110
eGFR [ml/(min*m^2^)]	94.14 ± 20.458	95.92 ± 24.399	103.23 ± 24.565	0.015	0.060	0.620
UA (μmol/L)	319.83 ± 79.948	330.08 ± 92.550	308.71 ± 78.652	0.390	0.038	0.218
TC (mmol/L)	4.25 ± 1.177	4.29 ± 1.108	4.46 ± 1.207	0.289	0.361	0.826
TG(mmol/L)	1.22 (0.93,1.62)	1.39 (0.99,1.89)	1.16 (0.74,1.82)	0.629	0.126	0.031
LDL-C(mmol/L)	2.43 ± 0.980	2.52 ± 0.897	2.65 ± 0.873	0.149	0.371	0.531
HDL-C(mmol/L)	1.20 ± 0.276	1.14 ± 0.283	1.22 ± 0.336	0.774	0.150	0.214
VLDL-C(mmol/L)	0.31 ± 0.148	0.25 ± 0.117	0.28 ± 0.153	0.299	0.140	0.008
SdLDL-C(mmol/L)	0.75 ± 0.359	0.71 ± 0.293	0.69 ± 0.305	0.372	0.739	0.531
HCY(μmol/L)	15.13 ± 16.055	17.19 ± 14.064	15.38 ± 6.679	0.913	0.364	0.403
ALT(U/L)	20.96 ± 20.491	18.97 ± 16.599	21.58 ± 15.841	0.838	0.327	0.520
AST(U/L)	24,41 ± 30.392	32.70 ± 65.358	30.92 ± 51.159	0.359	0.853	0.322
HbA1C(NGSP%)	6.52 ± 1.328	6.71 ± 1.709	6.56 ± 1.391	0.899	0.616	0.530
HbA1c(IFCC%)	47.78 ± 14.521	49.82 ± 18.673	48.15 ± 15.199	0.902	0.616	0.532
LP-PLA2(IU/L)	459.16 ± 115.44	451.88 ± 124.18	467.29 ± 111.13	0.702	0.478	0.744
CRP(mg/L)	1.00 (1.00,2.30)	1.00 (1.00,2.65)	1.00 (1.00,2.80)	0.930	0.748	0.757
TnI(ng/ml)	0.003 (0.00,0.01)	0.007 (0.00,0.04)	0.003 (0.00,0.02)	0.891	0.107	0.063
D-D(μg/ml)	0.30 (0.19,0.45)	0.32 (0.21,0.55)	0.29 (0.13,0.48)	0.689	0.355	0.209
BNP(pg/ml)	42.30 (24.55,67.15)	41.60 (26.00,85.70)	40.50 (25.00,40.50)	0.747	0.747	0.464
NT pro-BNP(pg/ml)	83.05 (29.10,149.33)	103.90 (53.60,183.40)	56.75 (28.00,177.23)	0.634	0.100	0.213
PSV(cm/s)	66.58 ± 14.053	66.21 ± 15.752	68.56 ± 16.860	0.440	0.363	0.876
EDV(cm/s)	19.42 ± 6.383	17.83 ± 5.403	20.85 ± 7.041	0.199	0.003	0.091
RI	0.71 ± 0.068	0.73 ± 0.059	0.69 ± 0.081	0.234	0.001	0.017
IMT(mm)	1.056 ± 0.152	1.22 ± 0.737	0.969 ± 0.206	0.006	0.008	0.067

^a^ Only the medium-high-risk plaque group was significantly different from the control group.

^b^ There were significant differences between the low-risk plaque group and the control group, and the medium-high-risk plaque group and the control group. But there was no significant difference between the low-risk plaque group and the medium-high-risk plaque group. *P1*, low-risk plaque group vs. control.

*P2*, medium-high-risk plaque group vs. control; *P3,* low-risk plaque group vs. medium-high-risk plaque group; low, low-risk plaque group; medium-high, medium-high-risk plaque group; Comparition of Male sex, Smoke, Hypertension, Diabetes between groups used the χ^2^ test; Comparition of TG, CRP, TnI, D-D, BNP, NT-proBNP between groups used the Rank sum test, and other projects’ comparition used t test; SBP, systolic blood pressure; DBP, diastolic blood pressure; PPD, Pulse pressure difference; MAP, mean arterial pressure; HR, heart rate; WBC, white blood cell; Hb, hemoglobin; PLT, Platelet; FBG, fasting blood glucose; Cys-C, Cystatin C; eGFR, estimated glomerular filtration rate; UA, uric acid; TC, total cholesterol; TG, triglycerides; LDL-C, low density lipoprotein cholesterol; HDL-C, high density lipoprotein cholesterol; VLDL-C, very low density lipoprotein cholesterol; SdLDL-C, small and low density lipoprotein cholesterol; AST, aspartate aminotransferase; ALT, alanine aminotransferase; HCY, homocysteine; HbA1c, glycosylated hemoglobin A1c; LP-PLA2, lipoprotein-associated phospholipase 2; CRP, C-reactive protein; TnI, troponin I; D-D, D-dimer; BNP, B-type brain natriuretic peptide; NT-proBNP, N-terminal pro-B-type natriuretic peptide; LVEF, left ventricular ejection fraction; PSV, peak systolic velocity; EDV, end-diastolic blood flow velocity; RI, Resistance index; IMT, intima-media thickness.

#### 3.3.2. Association between circSCMH1/miR-874 and plaque stability

**Figure 5:**
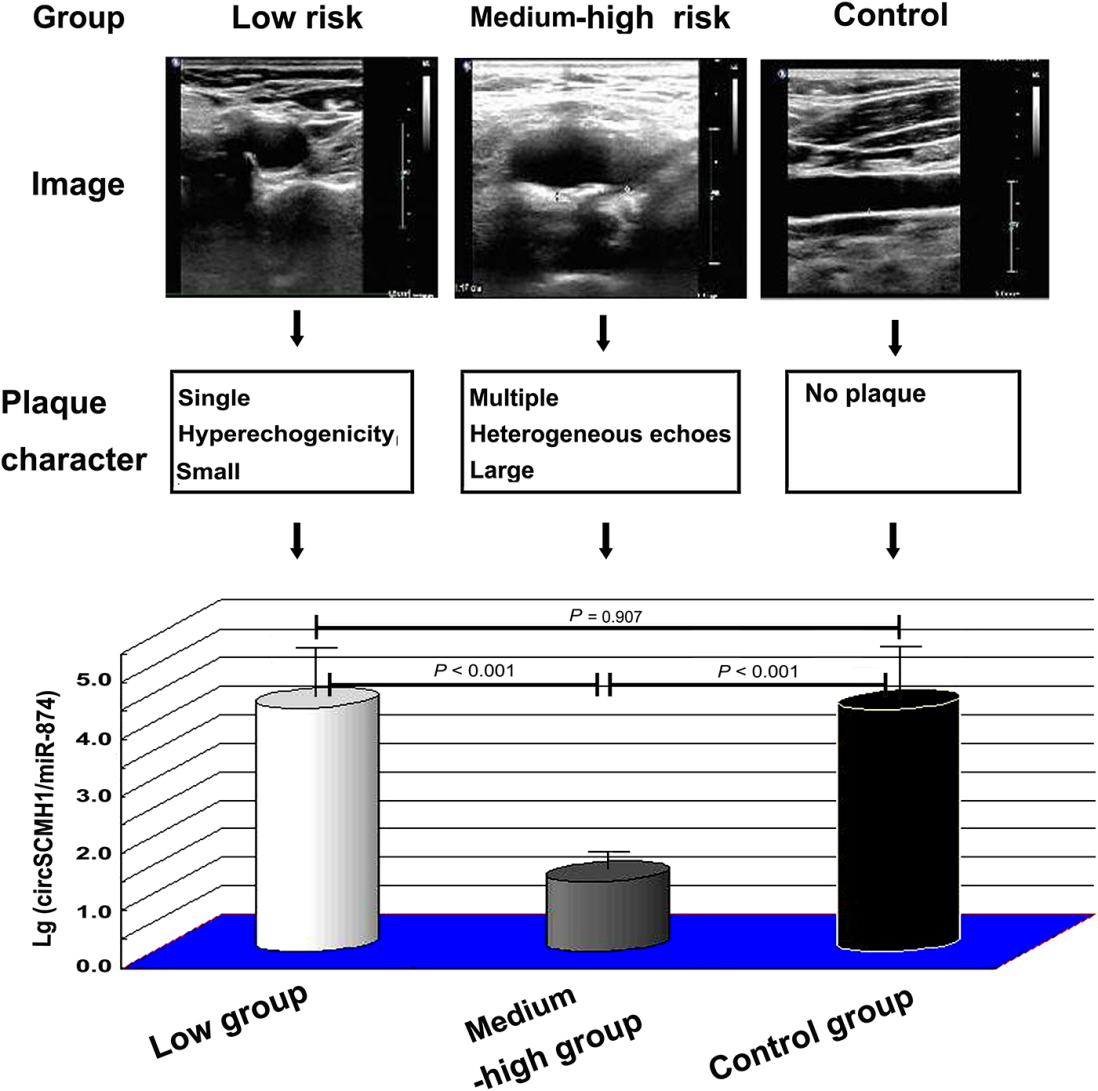
Typical carotid ultrasound images and lg (circSCMH1/miR-874) of patients in low-risk plaque, medium-high risk plaque and control groups. ANOVA test was performed between the three groups.

Compared to the control group, the lg (circSCMH1/miR-874) in the medium-high risk plaque group decreased, while there was no statistically significant difference between the low-risk plaque group and the control group (Figure 2B). Compared to the low-risk plaque group, the lg (circSCMH1/miR-874) in the medium-high-risk plaque group significantly decreased (Figure 2B, Figure 5). The detailed comparisons of typical carotid ultrasound images and lg (circSCMH1/miR-874) of the subjects in the three groups are presented in Figure 5.

#### 3.3.3. Analysis of risk factors affecting carotid plaque stability

Univariate and multivariate logistic regression analyses were performed with whether patients had medium-high-risk plaques as the dependent variable and some common risk factors in clinic among the three groups, as well as lg (circSCMH1/miR-874), as the independent variables (Table 7, Figure 3C). The single-factor logistic regression analysis showed that male, age, the history of smoking, hypertension and type 2 diabetes, SBP, PPD, PLT, Cys-C, UA, TG, VLDL-C, lg (circSCMH1/miR-874), EDV, RI, and IMT were significantly associated with the occurrence of medium-high-risk plaques (Table 7, Figure 3C). To control for potential confounding factors, multi-factor logistic regression analysis was performed and revealed that male, age, hypertension history, PPD, TG, lg (circSCMH1/miR-874), and IMT were independent risk factors for the occurrence of medium-high-risk plaques in the carotid artery. Notably, the risk of developing medium-high-risk plaques increased with the decrease of lg (circSCMH1/miR-874) (Table 7, Figure 3C).

**Table 7:** Logistic regression analysis stratified by medium-high risk carotid plaque.

Project	Univariate Logistic Regression			Multivariate Logistic Regression		
	OR	95% CI	*P*	OR	95% CI	*P*
Lg (circSCMH1/miR-874)	0.689	0.617–0.770	<0.001	0.577	0.455–0.732	<0.001
Male sex [case(%)]	2.671	1.543–4.622	<0.001	8.772	1.728–44.539	0.009
Age (year)	1.059	1.030–1.088	<0.001	1.157	1.056–1.267	0.002
Smoke [case (%)]	2.233	1.273–3.920	0.005	–	–	–
Hypertension [case(%)]	2.841	1.600–5.047	<0.001	1.115	1.022–1.614	0.011
Diabetes [case(%)]	2.218	1.220–4.033	0.009	–	–	–
SBP (mmHg)	1.017	1.007–1.027	<0.001	–	–	–
PPD (mmHg)	1.015	1.001–1.028	0.029	1.038	1.004–1.074	0.026
PLT (x109/L)	0.994	0.988–0.999	0.019	–	–	–
Cys-C (mg/L)	6.865	1.647–28.611	0.008	–	–	–
eGFR[ml/(min*m^2^)]	0.995	0.983–1.006	0.365	–	–	–
UA (μmol/L)	1.003	1.000–1.006	0.049	–	–	–
TG (mmol/L)	1.532	1.061–2.211	0.023	2.976	1.136–7.796	0.026
VLDL-C(mmol/L)	0.076	0.009–0.681	0.021	–	–	–
EDV(cm/s)	0.941	0.900–0.983	0.006	–	–	–
RI	526.869	10.995–25,246.808	0.002	–	–	–
IMT(mm)	155.479	18.941–1,276.282	<0.001	6.412	1.220–33.709	0.006

SBP, systolic blood pressure; PPD, pulse pressure difference; PLT, platelets; Cys-C, Cystatin C; eGFR, estimated glomerular filtration rate; UA, uric acid; TG, triglycerides; VLDL-C, very low density lipoprotein cholesterol; EDV, end-diastolic blood flow velocity; RI, Resistance index; IMT, intima-media thickness.

#### 3.3.4. Predictive value of different ratios for carotid plaques of medium-high risk

The diagnostic value of lg (circSCMH1/miR-874), NLR, MLR, PLR, TG/HDL-C ratio, monocyte/HDL-C ratio, and uric acid/neutrophil ratio for medium-high-risk plaques in patients were analyzed and compared the predictive value of these ratios using ROC curve analysis (Table 8, Figure 4C). The ROC curve analysis demonstrated that the area under the curve (AUC) of lg (circSCMH1/miR-874) was 0.815 (95% CI 0.753–0.877), with a sensitivity of 76.7%, specificity of 81.4%, and a cut-off value of 3.41. The AUC of uric acid/neutrophil ratio was 0.447 (95% CI 0.364–0.531), with a sensitivity of 41.1%, specificity of 47.1%, and a cut-off value of 92.01. The AUC of monocyte/HDL-C ratio was 0.571 (95% CI 0.488–0.654), with a sensitivity of 52.7%, specificity of 71.4%, and a cut-off value of 0.36. The AUC of TG/HDL-C ratio was 0.81 (95% CI 0.496–0.666), with a sensitivity of 84.5%, specificity of 34.3%, and a cut-off value of 1.69. The AUC of NLR was 0.559 (95% CI 0.477–0.642), with a sensitivity of 25.6%, specificity of 88.6%, and a cut-off value of 1.39. The AUC of PLR was 0.494 (95% CI 0.410–0.578), with a sensitivity of 49.6%, specificity of 57.1%, and a cut-off value of 120.48. The AUC of MLR was 0.573 (95% CI 0.489–0.657), with a sensitivity of 69.0%, specificity of 52.9%, and a cut-off value of 0.26. Therefore, lg (circSCMH1/miR-874) had good predictive value for medium-high-risk plaques in the carotid artery, and its comprehensive predictive value was better than other ratios (NLR, MLR, PLR, uric acid/neutrophil ratio, monocyte/HDL-C ratio, and TG/HDL-C ratio) mentioned above (Table 8, Figure 4C).

**Table 8:** Comparison of ROC curves for different ratios in predicting medium-high-risk plaque.

Project	AUC	Sensitivity	Specificity	Cut-off value	95% CI
Lg (circSCMH1/miR-874)	0.815	76.7%	81.4%	3.41	0.753–0.877
UA/neutrpphil ratio	0.447	41.1%	47.1%	92.01	0.364–0.531
monocyte/HDL-C ratio	0.571	52.7%	71.4%	0.36	0.488–0.654
TG/HDL-C ratio	0.581	84.5%	34.3%	1.69	0.496–0.666
NLR	0.559	25.6%	88.6%	1.39	0.477–0.642
PLR	0.494	49.6%	57.1%	120.48	0.410–0.578
MLR	0.573	69.0%	52.9%	0.26	0.489–0.657

AUC, area under curve; CI, confidence interval; UA, uric acid; NLR, neutrophil/lymphocyte ratio; PLR, platelet/lymphocyte ratio; MLR, monocyte/lymphocyte ratio.

## 4. Discussion

The purpose of this study is to determine whether circulating ncRNA can be used to identify patients who have recently experienced carotid artery or coronary artery related atherosclerotic cardio-cerebrovascular events, so as to predict and diagnose as early as possible, and start treatment earlier. The occurrence of coronary atherosclerotic heart disease and ischemic stroke is related to the rupture or instability of atherosclerotic plaques, while the unstable formation of atherosclerotic plaques is influenced by the combination of various pathological and risk factors. Many studies have explored its pathogenesis, such as the inflammation mediated by T lymphocytes mechanism (32), the plaque formation mechanism involved in vascular smooth muscle cells (33), and the inflammatory response mechanism involved in macrophages (34). The rupture of plaques is the main cause of myocardial infarction and can be caused and induced by various risk factors. Our research is precisely focused on the stability of plaques, aiming to provide a strong theoretical and clinical basis for the prevention and treatment of ischemic cardio-cerebrovascular diseases, reducing patient mortality and improving their prognosis.

In recent years, with the continuous advancement of imaging technology, various invasive and non-invasive imaging methods for the diagnosis of cardio-cerebrovascular disease, such as CCTA, B-mode ultrasound, contrast-enhanced ultrasound, high-resolution magnetic resonance imaging (MRI), IVUS, OCT, and positron emission tomography (PET), have been used to assess the stability of atherosclerotic plaques (16, 35). These methods can classify and evaluate various aspects of plaques, such as the size of the lipid necrotic core, thickness of the fibrous cap, and presence of neovascularization within the plaque (35). However, imaging inspection has many defects, such as high cost, radiation damage, contrast agent nephropathy or allergic reaction, induced heart failure or arrhythmia, large influence of operation imaging conditions, vascular damage, etc., which limit its application (16). Compared with imaging examinations, blood-related indicators have the advantages of low cost, simple and convenient operation, stability of quality control, minimal impact from operators, and better compliance of patients. Various serum biomarkers, such as FBG, HCY, Cys-C, blood lipid levels, interleukin-6 (IL-6), monocyte chemoattractant protein-1 (MCP-1), adiponectin and its derivatives, etc., have been discovered to be associated with plaque stability (36). Our study showed that compared with control group, patients of the ACS and SCAD groups had higher TG and lower HDL-C, which was consistent with previous studies. But the data showed the levels of TC and LDL-C of ACS and SCAD patients were lower than patients in control group. The FBG level of the patients in ACS groups showed no statistical difference compared with control group, neither than that of the patients in SCAD group. According to the risk level of carotid artery plaques, the blood lipid and blood glucose results obtained from the comparison between the high-risk group, low-risk group, and control group are similar to those of the three groups mentioned above. The results of our study not consistent with previous studies may be related to the use of hypoglycemic and lipid-lowering drugs by patients before admission, which could affect their own blood glucose and blood lipid levels. Similarly, the use of antihypertensive drugs can affect the patients' blood pressure, which could explain the difference between the comparison results of blood pressure-related indicators among the three groups in this study and previous studies. By contrast, it was consistent with the conclusions of most studies that multivariate analysis showed that male, history of diabetes, SBP and WBC were independent risk factors for ACS and AMI (Tables 2, 3, Figures 3A,B). This reflects that in addition to diabetes and hypertension being high risk factors for coronary events, inflammation is also an important factor involved. It is worth mentioning that UA is an independent risk factor for ACS (Table 2, Figure 3A). Currently, multiple studies have confirmed that UA has been identified as an important determinant of many different outcomes, such as all-cause and cardiovascular mortality, as well as cardiovascular events (mainly ACS) (37).

Based on the results of previous studies, composite indicators calculated from multiple indicators including NLR (10), MLR (11), PLR (14), TG/HDL-C (12), monocyte/ HDL-C (13), and uric acid/neutrophil ratios (38), have also been applied to plaque stability prediction. However, there is ongoing controversy over whether these biomarkers can be directly used in clinical work to assess the stability of carotid and coronary artery plaques for reasons of reliability, specificity or accuracy. Recently, ncRNAs have received much attention in molecular biology, especially circRNAs and miRNAs, which play an increasingly important role in evaluating and predicting disease development and prognosis (18, 26). At present, there are few reports on the correlation between circRNA/miRNA and arterial plaque stability (17). Previous studies have found that the expression of circSCMH1 was associated with ischemic stroke and miR-874 was related to myocardial infarction (18, 26). But no studies have confirmed whether the ratio of circSCMH1 to miR-874 is relevant to the instability of coronary and carotid artery plaques.

**Figure 6:**
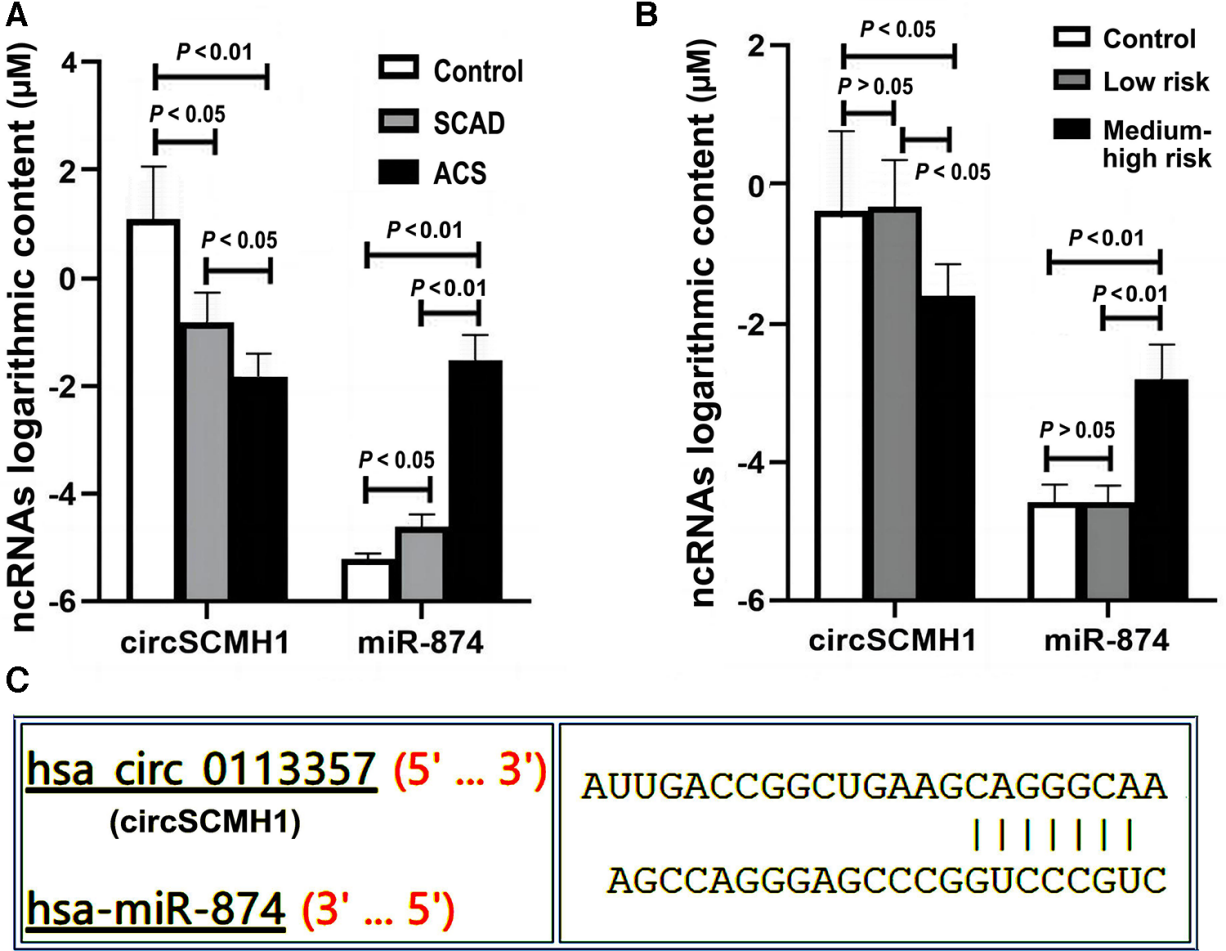
Expression of ncRNAs among different groups. Y-axis represents that the expression content of circSCMH1 and miR-874 in different groups is 10^Y^μM. ACS, acute coronary syndrome; SCAD, stable coronary artery disease; low-risk, low-risk plaque group, medium-high-risk plaque group; (**A**) expression content of circSCMH1 and miR-874 of ACS, SCAD and control groups; (**B**) expression content of circSCMH1 and miR-874 of low-risk plaque, medium-high risk plaque and control groups. Error bars represent standard deviation. ANOVA test was performed between the three groups.

Extracellular vesicles (EVs), which can usually be divided into three subgroups (exosomes, microvesicles, and apoptotic bodies), are structures released into their environment by all cells. Due to their lipid bilayer structure, EVs can be one of the naturally carrier vehicles that can transfer ncRNAs (including miRNAs, lncRNAs, and circRNAs) between cells and tissues (6, 8). From a structural and functional perspective, it is not very strict to distinguish exosomes from microvesicles. However, based on the reasons of detection accuracy, stability, and feasibility, we used exosomes instead of microvesicles in this study for the following reasons: Firstly, compared to microvesicles (with a diameter of approximately 100nm-1μm), the exosomes are more uniform (with a diameter of about 40–150 nm and the narrower particle size distribution range), indicating a smaller diameter dispersion (6, 8). Secondly, microvesicles are mainly formed by the budding of cell membranes such as platelets, red blood cells, and endothelial cells (6). Research has shown that the number of microcapsules released by red blood cells is easily influenced by environmental factors such as platelets are easily activated during sample collection and processing, and therefore cannot be stably released (39, 40). Thirdly, the frequency of cells releasing microvesicles under resting conditions is relatively low. When cell surface receptors are activated, cell apoptosis, or intracellular calcium ion concentration increases, the production of microvesicles will significantly increase (6). Fourthly, based on current research evidences, the most popular subgroup of EVs is exosomes. Its composition is the most complex, its functions are the most diverse, and its structure is relatively stable in all of extracellular vesicles. Therefore, exosomes may have the highest experimental and practical value, and may have been extensively studied and clinically applied. Fifthly, at present, the extraction and identification techniques for exosomes are more mature, which is more conducive to detection and more promising for clinical application in the future. The functions above make exosomes become our primary subject. Some miRNAs have been used to predict atherosclerotic plaque rupture and cardiovascular diseases such as AMI (8, 17). Not coincidentally, some previous studies have confirmed that circRNAs are expressed differently in various cardiovascular diseases in human serum exosomes (6, 9). CircRNA includes multiple miRNA binding sites, similar to sponge adsorbed water. CircRNA can specifically adsorb miRNA, playing the role of competitive endogenous RNAs (ceRNAs), isolating miRNA from target mRNA, thereby affecting the negative regulation of miRNA on target mRNA (28). In this study, we applied the method of bioinformatics analysis to search the literature and query the public RNA interaction database (Fig. http://www.mirdb.org, circinteractome.irp.nia.nih.gov, et al.) to search for target RNAs and speculate on possible interaction networks. Based on the results of bioinformatics scores, we selected three candidate miRNAs that interact with circSCMH1, including hsa-miR-615–5p, hsa-miR-645, and hsa-miR-874 (Supplementary Table S1). Then, we conducted preliminary experiments and found that hsa-miR-874 had better experimental results, so we ultimately chose hsa-miR-874 as the target miRNA. In this study, it was found that there were significant differences in peripheral blood cirSCMH1 and miR-874 contents between ACS, ACAD and the control group, as well as between the medium-high risk plaque group, the low-risk plaque group and the control group (Figures 6A,B), indicating that the ratio of circRNAs and miRNAs may be biomarkers for cardiovascular diseases. Moreover, circSCMH1 and miR-874 exhibited opposite trends (Figures 6A,B). Based on bioinformatics analysis, there actually are interaction sites between circRNAs and miRNAs (Figure 6C).

This study found that the logarithm of the ratio of circSCMH1 to miR-874 [lg (circSCMH1/miR-874)] could better reflect the comparability of ncRNA among SCAD, ACS and control group than that of single circSCMH1 or miR-874, and can better predict coronary atherosclerotic lesions, the stability and instability of atherosclerotic plaque, and medium-high risk and low risk (Figures 2–5). As shown in Figure 2A and Supplementary Figure S2, the ACS group had a lower lg (circSCMH1/miR-874) than the SCAD and control groups. This finding is consistent with previous studies on changes in circSCMH1 and miR-874 levels during atherosclerotic lesion (18, 27, 28). Multivariate logistic regression analysis showed that lg (circSCMH1/miR-874) was an independent risk factor for ACS, AMI, and the carotid artery plaques of medium-high risk, regardless of whether coronary lesion degree was used as the dependent variable or the medium-high risk of carotid artery plaques was used as the dependent variable (all *OR* < 1, all *P* < 0.05) (Tables 1, 2, 6). That is, with the decrease of lg (circSCMH1/ miR-874), patients have an increased risk of developing ACS, AMI, and AMI (Tables 2, 3). For each additional unit of Lg (circSCMH1/miR-874), the risk of developing ACS is reduced by 0.240 times and the risk of developing AMI is reduced by 0.305 times (Tables 2, 3; Figures 3A,B).

Figures 4A,B displayed that lg (circSCMH1/miR-874) has good predictive value for ACS and AMI, with cut-off values of 1.52 and 1.38, respectively, and has high specificity and sensitivity. Additionally, when comparing the receiver operating characteristic (ROC), sensitivity, and specificity of lg (circSCMH1/miR-874) with common prediction ratios such as PLR, NLR, MLR, TG/HDL-C, monocyte/HDL-C, and uric acid/neutrophil ratios, as a result, it was found that lg (circSCMH1/miro-874) had the best comprehensive prediction value (Figures 4A, 6B). Likewise, lg (circSCMH1/miR-874) could be a biological indicator for evaluating the stability of carotid plaques of medium-high risk rather than low-risk (Figure 2B). As expected, lg (circSCMH1/miR-874), as an independent risk factor for moderate-high risk plaque, has a high predictive value for moderate-high risk plaque. Each additional unit was associated with a 0.577 fold reduction in the risk of having moderate-high risk carotid plaque (Table 7, Figure 3C). It is worth mentioning that in addition to male, age, blood pressure and intimal artery thickness, PPD and TG of patients are independent risk factors for moderate-high risk plaque. This may be related to the higher PPD value due to the older age, obvious arteriosclerosis and poor arterial elasticity in the medium-high-risk plaque group. In this study, TG truly reflects the adverse effects on atherosclerotic plaque, and the elevation of TG is particularly prominent in dyslipidemia of Chinese people. Unlike LDL-C, TG is less affected by statins. As shown in the Table 8, lg (circSCMH1/miR-874) with the cutoff value of 3.41 had higher sensitivity and specificity and could be used as a screening indicator for preliminary evaluation of the carotid plaque with medium-high risk (Table 8).

So far, the evaluation of arterial plaque stability in clinical practice mainly relies on imaging examination, which may be invasive, costly, potentially allergic, contrast nephrotoxic, and dependent on the operator's level of expertise as mentioned above. Instead, as a new index to predict the stability of carotid and coronary plaque, the detection of lg (circSCMH1/miR-874) in this study only requires one venous blood sample, which is convenient, economical, safe and more easily accepted by patients. Moreover, as an objective indicator, it was less affected by the subjectivity of the tester. Therefore, lg (circSCMH1/miR-874), as a preliminary screening indicator for high-risk atherosclerotic plaque, has significant advantages in clinical practice. Since exosomes are natural ncRNA carriers, the detection of ncRNA content in exosomes can more accurately reflect the accuracy of detection results. Furthermore, compared to the detection of single miRNAs or circRNAs, the calculation of their ratio is less affected by the total RNA content of peripheral blood exosomes from individual patients, and therefore more accurate. In comparison to other ratios, the comprehensive sensitivity and specificity of the lg (circSCMH1/miR-874) ratio is higher (Tables 4, 5, 8; Figure 4). Moreover, it is less influenced by factors such as the patient's own diet, allergic constitution, liver and kidney function, making it of greater significance for the preliminary screening of unstable plaques in the carotid and coronary arteries.

## 5. Limitations

Firstly, a considerable number of patients with ACS belong to coronary microcirculation disorder without obvious coronary plaque. Whether circSCMH1/miR-874 is related to this group of people needs further research. Secondly, for evaluating the stability of carotid atherosclerotic plaques, the method used in this study to assess risk and stratify risk levels using carotid ultrasound lacks support from other imaging evaluation methods, such as IVUS, calcium scoring based on CTA, and vulnerability scoring based on OCT. Therefore, further studies are needed to explore whether combining this method with related techniques such as imaging can improve the detection rate of unstable plaques. Thirdly, traditional factors that contribute to the occurrence and development of atherosclerotic plaques, such as sex, age, history of hypertension, history of diabetes, smoking history, FBG, and blood lipid levels, are all shown to be related to the occurrence of ACS, AMI, and carotid plaques with high risk, according to single-factor logistic analysis, which is consistent with previous research results. However, in further multivariate logistic regression analysis, FBG and LDL-C levels are often excluded from independent risk factors, which is contrary to previous research. This may be due to the fact that many patients in the study were already taking hypoglycemic, antihypertensive, and lipid-lowering drugs before enrollment, which may have influenced their FBG and blood lipid levels after enrollment and may have affected the statistical results. Therefore, it is necessary to further investigate the original data of relevant indicators of the enrolled patients before medication. However, it is difficult to obtain or verify the original data of some patients before their onset. Additionally, this study only included patients from the Second Hospital of Shandong University. The source of patients was relatively single and the number of samples was small. Therefore, further multicenter and large sample Prospective cohort studies are needed to further explore the relationship between lg (circSCMH1/miR-874) and plaque stability and coronary events. Finally, our cohort only comprises Chinese individuals, thus it may limit the generalizability of our findings to other ethnicities.

## 6. Conclusion

Lg (circSCMH1/miR-874) has an excellent predictive effect on medium-high-risk carotid plaques, ACS, and AMI, and can be used as a biomarker for assisting in the initial screening of high-risk cardio-cerebrovascular events in patients. As a surrogate for predicting the stability of arterial plaque, lg (circSCMH1/miR-874) performs better than other previously reported predictors in effectiveness, sensitivity and specificity, such as NLR, MLR, PLR, TG/HDL-C ratio, Monocyte/HDL-C ratio, and blood uric acid/neutrophil ratio.

## Data Availability

The datasets presented in this study can be found in online repositories. The names of the repository/repositories and accession number(s) can be found below: NM_001031694 and NM 001198, https://www.ncbi.nlm.nih.gov/nuccore.
