# Supplementary material for: Exosomal circSCMH1/miR-874 ratio in serum to predict carotid and coronary plaque stability
Source: Front Cardiovasc Med. 2023 Dec 11;10:1277427. doi: 10.3389/fcvm.2023.1277427 (PMC10750349; doi:10.3389/fcvm.2023.1277427)
Supplement: Supplementary file 1 [file Table1.doc]

**Tables**

**Table S1. Target miRNA prediction**

| miRNA | CircSCMH1 (Top) - miRNA (Bottom) pairing | bioinformatics score |
| --- | --- | --- |
| hsa-miR-874(5’→3’) | AUUGACCGGCUGAAGCAGGGCAA  | | | | | | |  AGCCAGGGAGCCCGGUCCCGUC | 99 |
| hsa-miR-615-5p(5’→3’) | GCUGAUCCUCAGCUUGGACCCCA  | | | | | | |  CUAGGCUCGUGGCCCCUGGGGG | 98 |
| [hsa-miR-645](http://www.mirbase.org/cgi-bin/mirna_entry.pl?acc=hsa-miR-645)(5’→3’) | UGAACCAGGAGAGGCAGCCUAGA  | | | | | | |  AGUCGUCAUGGUCGGAUCU | 98 |
| [hsa-miR-146b-3p](http://www.mirbase.org/cgi-bin/mirna_entry.pl?acc=hsa-miR-146b-3p)(5’→3’) | AUUGACCGGCUGAAG-----CAGGGCAA  | | | | | | | | |  GGUCUUGACUCAGGUGUCCCGU | 93 |
| [hsa-miR-217](http://www.mirbase.org/cgi-bin/mirna_entry.pl?acc=hsa-miR-217)(5’→3’) | ACAGUCGAGGAUGUGAUGCAGUU  | | | | | | |  AGGUUAGUCAAGGACUACGUCAU | 85 |
| [hsa-miR-668](http://www.mirbase.org/cgi-bin/mirna_entry.pl?acc=hsa-miR-668)(5’→3’) | CUGCUGCUGCUGCGCAGUGACAU  | | | | | | |  CAUCACCCGGCUCGGCUCACUGU | 78 |
| [hsa-miR-766](http://www.mirbase.org/cgi-bin/mirna_entry.pl?acc=hsa-miR-766)(5’→3’) | GGUCGGACCGAUACCUGGAGAG  | | | | | |  CGACUCCGACACCCCGACCUCA | 70 |

The Prediction of miRNAs and bioinformatics score were obtained by query through the TargetScan website (https://circinteractome.irp.nia.nih.gov/).

**Table S2. ncRNAs gene information and PCR primers**

| **ncRNA name** | **GenBank Accession** | **Gene**  **Symbol** | **Primer** | **Primer Sequence (5’→3’)** |
| --- | --- | --- | --- | --- |
| hsa-circ-0113357 | NM_001031694 | SCMH1 | circSCMH1-172F primer | GCTCTCCTACACATTGACCG |
|  |  |  | circSCMH1-172R primer | GACAAACTGCATCACATCCTCG |
| hsa-miR-874-3p | NM 001198 | PRDM1 | hsa-miR-874 F primer | ATTATTTATCTGCCCTGGCC |
|  |  |  | hsa-miR-874 R primer | TATGGTTGTTCACGACTCCTTCAC |

Hsa-circ-0113357 and hsa-miR-874-3p are referred to as circSCMH1 and miR-874 respectively in this study

**Legends of figures**

**Figure S1. Identification of exosomes.**

The results of exosomes electron microscopy analysis for groups ACS, SCAD, and control are as follows: A, results for ACS group; B, results for SCAD group; C, results for control group. D, exosomes were mostly between 30 and 150 nm in diameter; E, the WB results of TSG101; F, the WB results of CD81; The WB results, from left to right, are ACS, SCAD, and control group.

**Figure S2. Typical CTA images and lg (circSCMH1/miR-874) of ACS, SCAD and control patients.**

Select a typical coronary CTA image from one patient in each of the three groups. ACS, acute coronary syndrome; SCAD, stable coronary artery disease; CTA, computer tomography angiography. VRT, volume rendering technique; MIP, maximum intensity projection; CPR, curve planar reconstruction.
