# Supplementary figures and images for: Exosomal circSCMH1/miR-874 ratio in serum to predict carotid and coronary plaque stability
Source: Front Cardiovasc Med. 2023 Dec 11;10:1277427. doi: 10.3389/fcvm.2023.1277427 (PMC10750349; doi:10.3389/fcvm.2023.1277427)

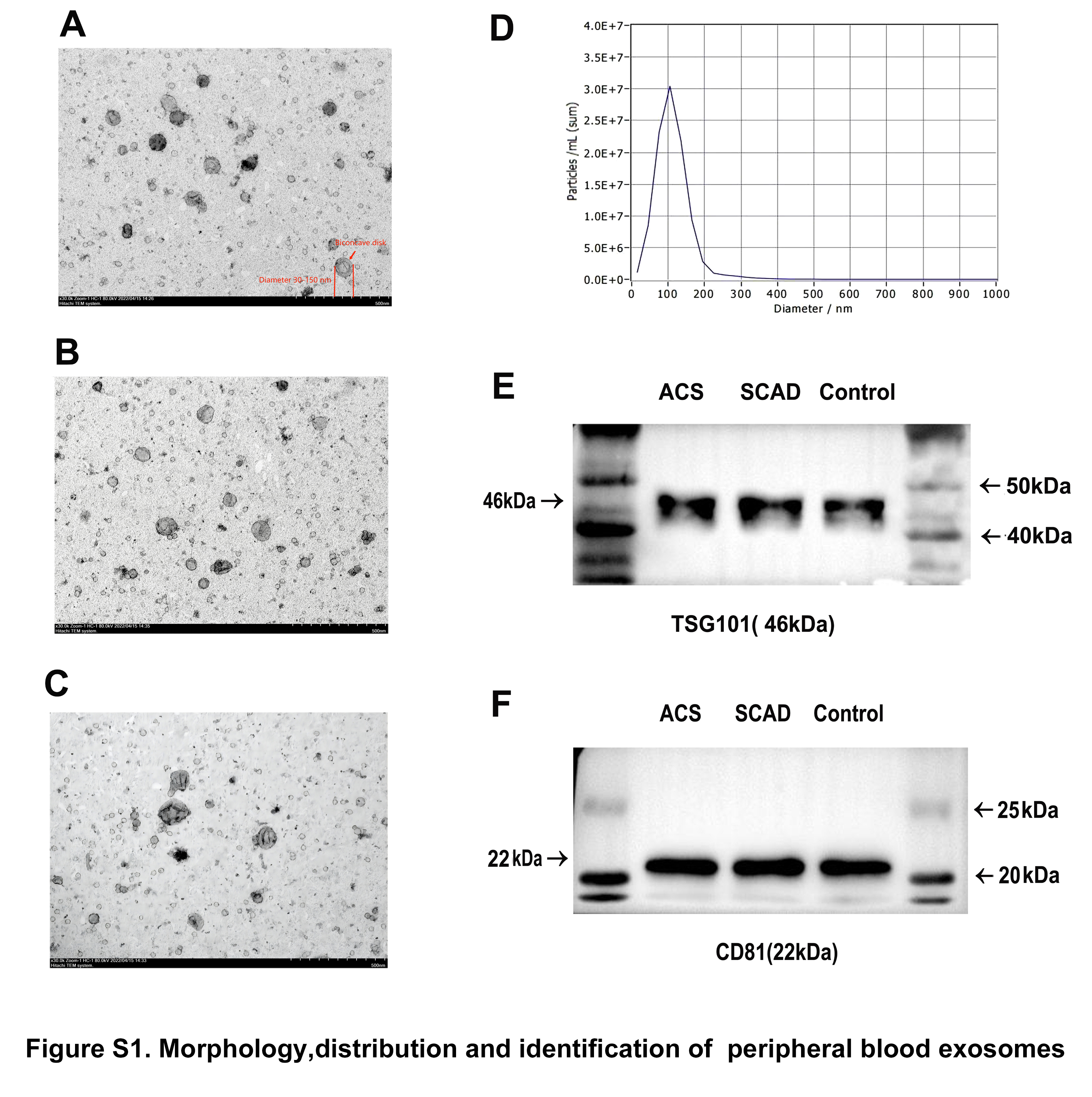

Supplement: Supplementary file 2 [file Image1.jpeg]

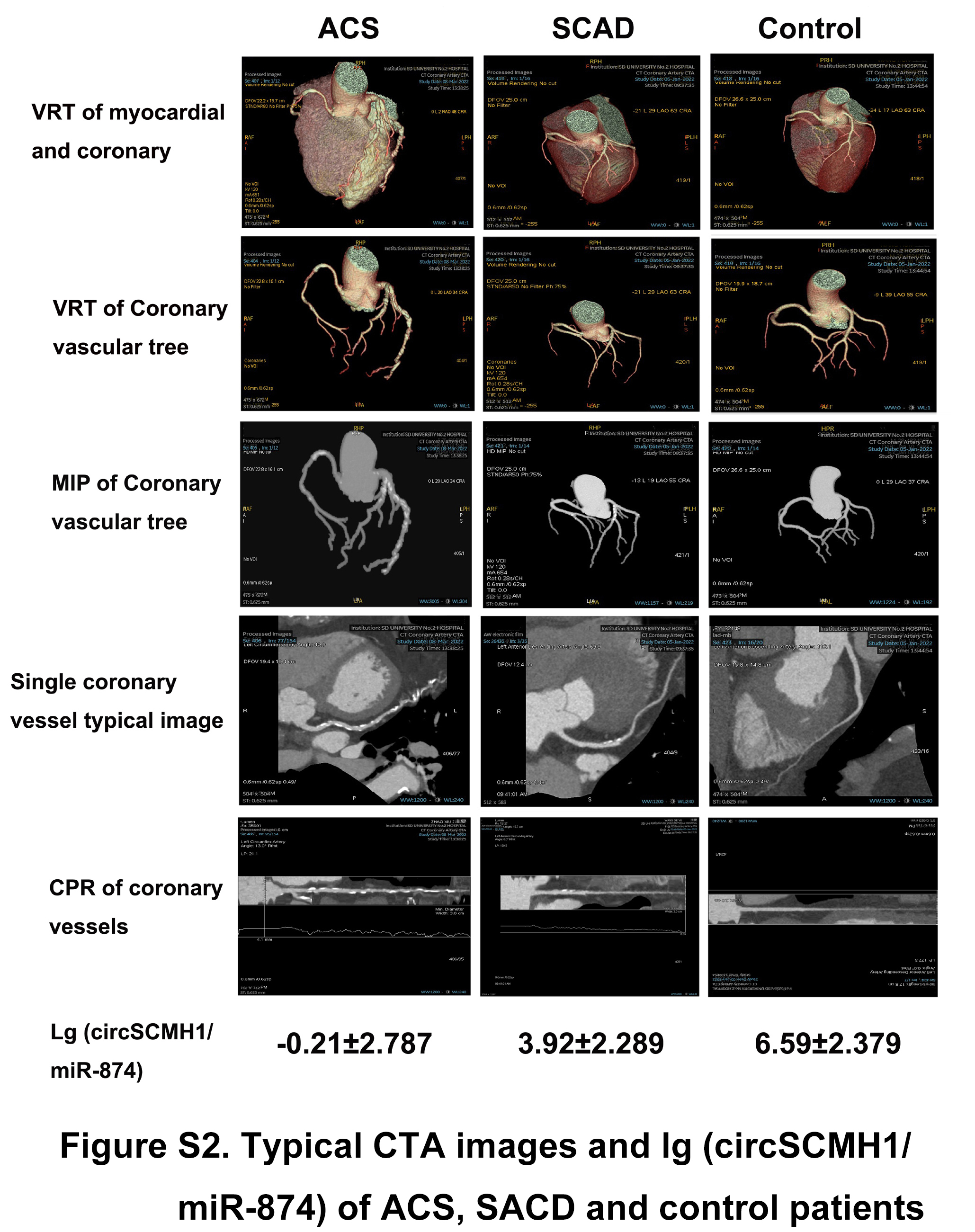

Supplement: Supplementary file 3 [file Image2.jpeg]
